# Safety Evaluation of Repeated Application of Polymeric Microarray Patches in Miniature Pigs

**DOI:** 10.1002/adhm.202501512

**Published:** 2025-06-17

**Authors:** Qonita Kurnia Anjani, Aaron R. J. Hutton, Peter E. McKenna, Eneko Larrañeta, Ryan F. Donnelly

**Affiliations:** ^1^ School of Pharmacy, Queen's University Belfast Medical Biology Centre 97 Lisburn Road Belfast BT9 7BL UK; ^2^ School of Pharmacy and Pharmaceutical Sciences Ulster University Pharmacy Building, Cromore Rd Coleraine BT52 1SA UK

**Keywords:** dissolving MAPs, hydrogel‐forming MAPs, implantable MAPs, miniature pigs, repeated application, safety

## Abstract

The safety of repeated microarray patch (MAP) application is crucial for its development as an innovative drug delivery platform. This study is the first to assess the safety of repeated applications of hydrogel‐forming, dissolving, and implantable MAPs over four weeks using miniature pigs, an industry‐standard dermatological model with human‐like skin structure and physiological responses. Uniform MAPs are successfully manufactured, with application forces of 32 N/array resulting in less than 15% needle height reduction. ≈80% of the needle length penetrated Parafilm layers, while 40–60% penetrated excised porcine skin. Repeated MAP applications do not compromise skin barrier function, as confirmed by transepidermal water loss measurements, and caused no adverse skin reactions per modified Draize test results. Systemic safety assessments revealed no significant immune responses, allergic reactions, infections, or inflammatory markers (TNF‐α, IgE, IgG, CRP, and IL‐1β) between day 0 and day 28. No weight loss, infection signs, kidney toxicity, or clinically relevant hematological or biochemical changes are observed. Histopathological evaluations confirmed the absence of lesions or adverse effects. These findings establish the safety of repeated hydrogel‐forming, dissolving, and implantable MAP applications, supporting their potential for safe, effective drug delivery and facilitating their translation from preclinical models to human clinical trials.

## Graphical abstract

This study demonstrates the safety of repeated application of three types of microarray patches (MAPs), hydrogelforming, dissolving, and implantable, over four weeks in miniature pigs. No adverse skin or systemic effects were observed, supporting the clinical potential of MAPs as safe and effective drug delivery platforms.

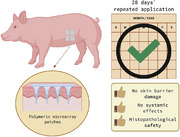

## 1 Introduction

Microarray patches (MAPs) are an innovative drug delivery system that provides a minimally invasive and efficient alternative to conventional administration methods such as injections or oral dosing.^[^ ^1^, ^2^, ^3^, ^4^ ^]^ By employing micron‐sized needles that penetrate the *stratum corneum*, MAPs enable the painless delivery of therapeutic agents into the epidermis or dermis, allowing for localized effects (e.g., immune cell‐targeted vaccination) or systemic drug delivery.^[^ ^5^ ^]^ Their advantages, including enhanced patient compliance, self‐administration capability, and reduced reliance on healthcare professionals, make MAPs particularly valuable in low‐resource settings.^[^ ^6^ ^]^ Previous studies have demonstrated their versatility across various applications, including vaccine delivery, chronic disease management, and diagnostics, positioning MAPs as a transformative tool in modern healthcare.^[^ ^7^, ^8^, ^9^, ^10^, ^11^ ^]^

Among MAP technologies, polymeric MAPs have garnered significant attention due to their ease of manufacture, biocompatibility, mechanical strength, and adaptability. Hydrogel‐forming MAPs swell upon contact with interstitial fluid, creating a diffusion pathway for drug delivery without leaving measurable polymer residues in the skin.^[^ ^12^, ^13^ ^]^ Dissolving MAPs disintegrate upon application, releasing their payloads, while implantable MAPs provide sustained drug release for extended therapeutic effects.^[^ ^14^, ^15^, ^16^, ^17^ ^]^

In certain disease conditions, patients may need to apply MAPs repeatedly on a daily, weekly, or monthly basis, a scenario referred to as “repeated” application. The safety of repeated MAP use depends on several critical factors. One primary concern is the potential disruption of the skin barrier, particularly the *stratum corneum* and epidermis.^[^ ^18^ ^]^ Another consideration is systemic immune activation, as repeated MAP application could trigger localized or systemic inflammation. Additionally, kidney toxicity is a potential risk with polymeric MAPs, as polymer fragments with molecular weights exceeding the renal filtration threshold may accumulate in the kidneys, potentially impairing renal function. Monitoring parameters such as protein levels, creatinine, and urine‐specific gravity is essential for assessing nephrotoxicity and overall kidney health.

While previous studies have investigated the safety of repeated MAP application, most have been conducted in academic settings with limited translational applicability. Vicente‐Perez *et al.* reported that repeated application of hydrogel‐forming MAPs for three weeks and dissolving MAPs for four weeks in mice did not significantly alter transepidermal water loss (TEWL) or biomarkers of infection, immunity, or inflammation.^[^ ^19^ ^]^ Similarly, Al‐Kasasbeh *et al.* demonstrated that repeated hydrogel‐forming MAP application in human volunteers did not cause prolonged skin reactions or barrier disruption, with biomarker concentrations remaining within normal ranges, indicating no systemic effects.^[^ ^20^ ^]^ However, to date, no comprehensive study has evaluated the repeated application of hydrogel‐forming, dissolving, and implantable MAPs across all safety aspects in a Good Laboratory Practice (GLP) model adhering to industrial standards.

To address this gap, the present study was conducted in full compliance with the OECD Principles of Good Laboratory Practice (revised 1997, C(97)186/Final) and applicable EU regulations (Directive 1999/11/CE and Real Decreto 1369/2000). The protocol was approved by the ethical committee of Specific Pig (Specipig) S.L., and all procedures were carried out at Specipig's GLP‐certified facility in Barcelona, Spain. The study was also subject to routine inspections to ensure adherence to GLP through both facility‐level and process‐based evaluations. This rigorous regulatory framework enhances the translational relevance of our findings and supports their potential application in clinical development.

Miniature pigs are an ideal preclinical model for assessing MAP safety due to their close resemblance to human skin in terms of epidermal thickness, dermal‐epidermal junction structure, and lipid composition.^[^ ^21^ ^]^ These similarities make them particularly suited for evaluating the localized effects of MAPs on skin integrity and barrier function. Furthermore, the systemic physiology of miniature pigs allows for robust assessments of immune and renal responses under conditions mimicking human use.^[^ ^22^, ^23^ ^]^ This model bridges the gap between preclinical and clinical studies, providing valuable insights into MAP safety and paving the way for human trials.

In this study, we conducted, for the first time, a comprehensive evaluation of the safety of repeated application of three types of polymeric MAPs (hydrogel‐forming, dissolving, and implantable) over a 28‐day period in miniature pigs under GLP industrial standard. This investigation addresses critical knowledge gaps regarding the long‐term safety of MAPs, providing insights into both localized and systemic effects. The findings have significant implications for the development of MAP‐based therapies, particularly for drug delivery requiring repeated applications. From a regulatory perspective, this study contributes to establishing safety benchmarks for preclinical evaluations, facilitating the translation of MAP technology from animal models to human clinical trials.

## 2 Results and Discussion

### 2.1 Preparation and Characterisation of Polymeric MAPs

In this study, three types of polymeric MAPs were prepared, namely hydrogel‐forming, dissolving, and implantable MAPs, all without active compounds to evaluate the safety of repeated applications. Previous studies from other groups have focused on single MAP applications.^[^ ^24^, ^25^ ^]^ These studies are important, but they obviate the importance of repeated applications. Drug delivery applications using MAPs will likely require repeated applications by the patient.

This study focused on the three primary types of MAPs commonly described in the literature for drug delivery. The proposed systems cater to varying treatment durations: dissolving MAPs are typically used for short‐term drug delivery, implantable MAPs support sustained drug release over extended periods (weekly or monthly), and hydrogel‐forming MAPs can be adapted for either short‐term or prolonged drug administration by modifying the drug reservoir and hydrogel matrix. Depending on the specific application, these MAPs may remain in place for up to five days, as demonstrated in our previous studies.^[^ ^26^, ^27^ ^]^

Once inserted, MAPs create microchannels that bypass the *stratum corneum*, the primary barrier to transdermal drug delivery. The mechanism of drug release varies by MAP type. Hydrogel‐forming MAPs rapidly swell upon contact with interstitial fluid, creating a hydrated, continuous channel between the drug reservoir and the dermal microcirculation, enabling controlled diffusion over time.^[^ ^13^, ^28^, ^29^ ^]^ In contrast, dissolving MAPs are designed to fully dissolve within the viable epidermis and upper dermis, releasing their drug payload directly into these layers.^[^ ^30^ ^]^ Implantable MAPs deposit solid drug‐loaded tips that remain embedded in the skin and degrade over time, providing a sustained release profile.^[^ ^7^, ^17^, ^26^ ^]^ Once delivered, drugs may exert local effects or be absorbed into systemic circulation depending on their physicochemical properties, molecular weight, and the local vascularisation of the application site. This combination of precise skin penetration, material responsiveness, and programmable drug release kinetics underpins the versatility of MAP technology across a range of therapeutic needs.

**Figure 1:**
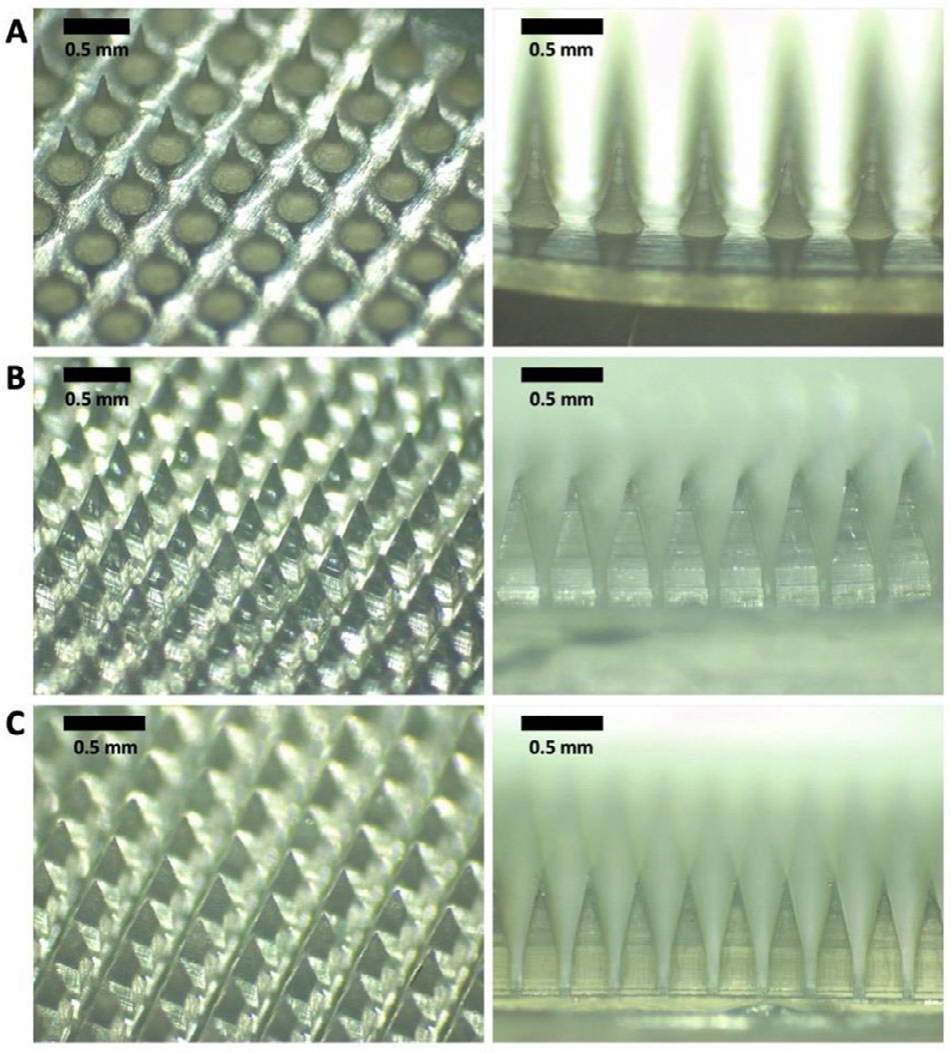
Microscopic images of different types of blank MAP systems. (A) hydrogel‐forming MAPs, (B) dissolving MAPs, and (C) implantable MAPs.

The hydrogel‐forming MAPs, composed of Gantrez S‐97 and PEG, featured conical needles with a height of 600 µm (**Figure** **1**A). In contrast, the dissolving and implantable MAPs had cuboidal bases with pyramidal tips (Figure 1B,C). These distinct geometries were selected based on their intended functions. Hydrogel‐forming MAPs are designed to penetrate the *stratum corneum* and epidermis, absorb interstitial fluid in the upper dermis, and swell, thereby facilitating drug diffusion from a reservoir for systemic absorption.^[^ ^12^, ^13^, ^27^, ^31^, ^32^, ^33^ ^]^ Conversely, dissolving and implantable MAPs are optimized to deposit drugs directly into viable skin layers, making them suitable for immediate local, systemic, or long‐acting drug delivery.^[^ ^34^, ^35^, ^36^, ^37^, ^38^ ^]^ The pyramidal needle tips enable high drug loading, ensuring efficient delivery to the dermal microcirculation beneath the *stratum corneum*.^[^ ^34^ ^]^ Poly(dimethylsiloxane) molds were used to produce the cuboidal‐pyramidal needle geometry, chosen for its superior insertion and drug delivery efficiency compared to other shapes.^[^ ^39^ ^]^ The resulting MAPs exhibited uniform, sharp needle structures (Figure 1), which were further evaluated for mechanical and insertion performance using Parafilm layers and excised porcine skin before application to miniature pigs.

**Figure 2:**
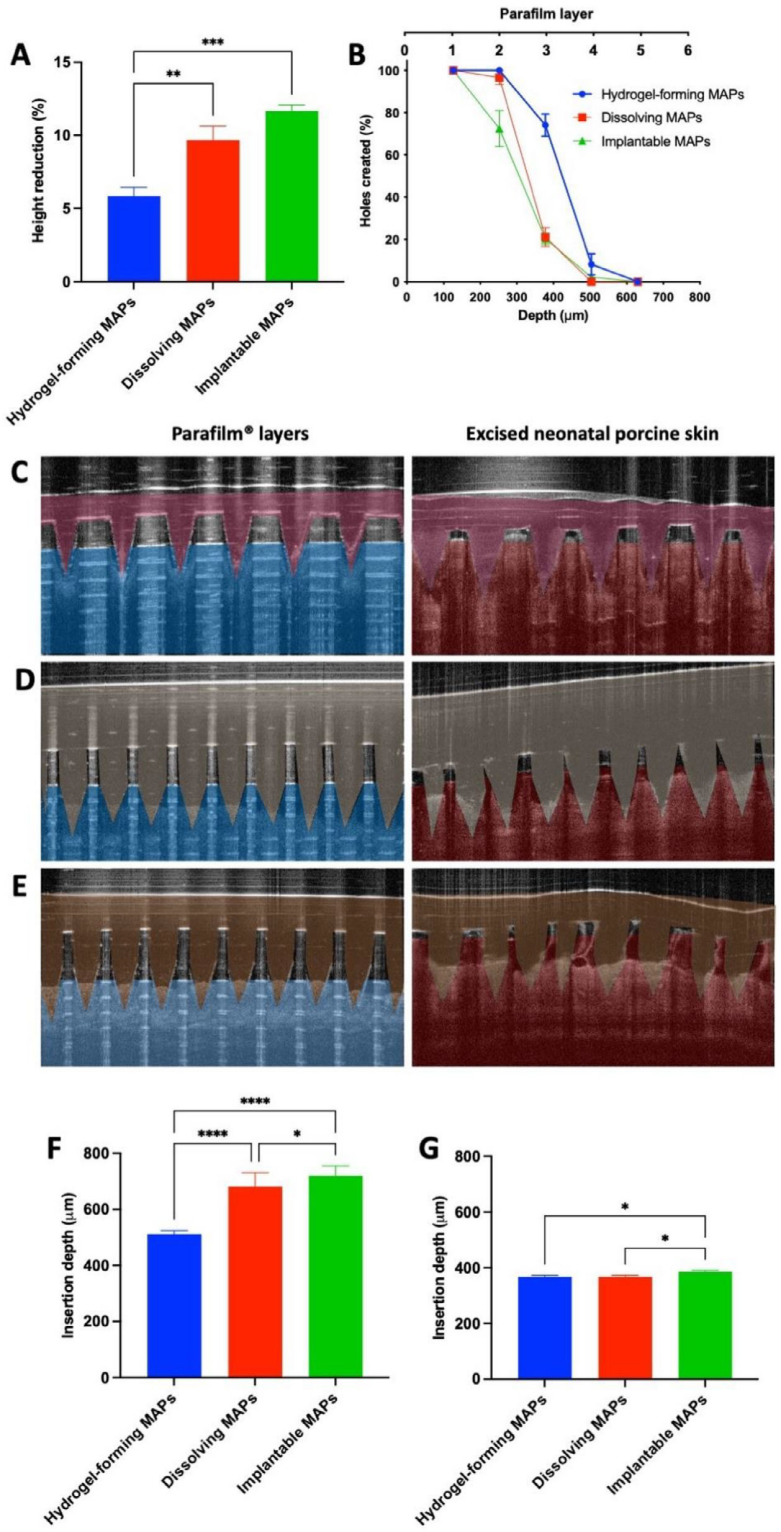
(A) Percentage reduction in needle height after compression with a 32 N force for 30 s (means + SD, n  =  20). (B) Insertion capabilities of MAPs into stacked Parafilm layers, measured by the number of holes created in each layer (means ± SD, n  =  3). Representative false color OCT images of MAP insertion into Parafilm and excised porcine skin for (C) hydrogel‐forming MAPs, (D) dissolving MAPs, and (E) implantable MAPs. Insertion depth (µm) and percentage insertion efficiency of (F) hydrogel‐forming MAPs, (G) dissolving MAPs, and implantable MAPs into Parafilm M and full‐thickness porcine skin, as measured by OCT (means + SD, n  =  20).

Compression tests (32 N for 30 s) showed that all MAP types exhibited less than a 15% reduction in needle height, with hydrogel‐forming MAPs demonstrating significantly lower height reduction than dissolving and implantable MAPs (*p* < 0.05, **Figure** **2**A). These findings align with previous reports on the mechanical properties of hydrogel‐forming MAPs,^[^ ^13^, ^27^, ^32^, ^40^, ^41^ ^]^ dissolving MAPs,^[^ ^30^, ^42^, ^43^, ^44^, ^45^ ^]^ and implantable MAPs.^[^ ^17^ ^]^ The superior mechanical properties of hydrogel‐forming MAPs is attributed to the crosslinking between Gantrez S‐97 and PEG during heating at 80°C,^[^ ^13^, ^27^ ^]^ ensuring structural integrity under the forces required for skin insertion. It is important to note that this test primarily serves as a comparative tool to assess MAP mechanical integrity. While applying force against an aluminum block does not replicate real‐world application conditions, it provides a standardized method to compare formulations and eliminate those with insufficient strength.

Insertion studies using Parafilm layers and excised porcine skin (Figure 2B) demonstrated that hydrogel‐forming and dissolving MAPs created 100% perforation in the first two layers (≈250 µm depth), while implantable MAPs created ≈72% perforation in the same layers. Hydrogel‐forming MAPs remained superior in the third layer (≈375 µm depth), achieving ≈74% perforation compared to ≈20% for the other MAPs. The material properties and needle geometry play a critical role in MAP insertion efficiency.^[^ ^46^, ^47^ ^]^ The conical design of hydrogel‐forming MAPs resulted in deeper insertion than the cuboidal design, likely due to the crosslinked polymer structure, as demonstrated in previous studies.^[^ ^13^, ^41^ ^]^ Despite having the same design, implantable MAPs showed lower insertion efficiency than dissolving MAPs, likely due to the presence of PLGA tips.

Optical coherence tomography (OCT) imaging (Figure 2C–E) further confirmed the insertion profiles, revealing gaps between the MAP baseplate and the Parafilm or porcine skin surface, suggesting incomplete penetration. This incomplete insertion in the skin is likely caused by elastic recoil after pressure release.^[^ ^48^ ^]^ ImageJ analysis estimated insertion depths, showing that ≈80% of the needle length penetrated Parafilm layers, while 40–60% penetrated excised porcine skin (Figure 4F,G). The variation in penetration may be due to differences in material properties and elasticity between Parafilm and full‐thickness neonatal porcine skin. To preserve skin integrity during the study, the porcine skin was equilibrated in PBS (pH 7.4) before testing. The presence of saline likely acted as a lubricant, facilitating deeper needle insertion compared to Parafilm.^[^ ^45^ ^]^ In vivo, MAPs will be secured with adhesives to ensure consistent skin contact throughout the study. These results align with previous findings, confirming the ability of these MAPs to effectively penetrate the *stratum corneum* and epidermis, the primary barriers to transdermal drug delivery.

### 2.2 Clinical Scoring of Skin Irritation and Erythema

**Figure 3:**
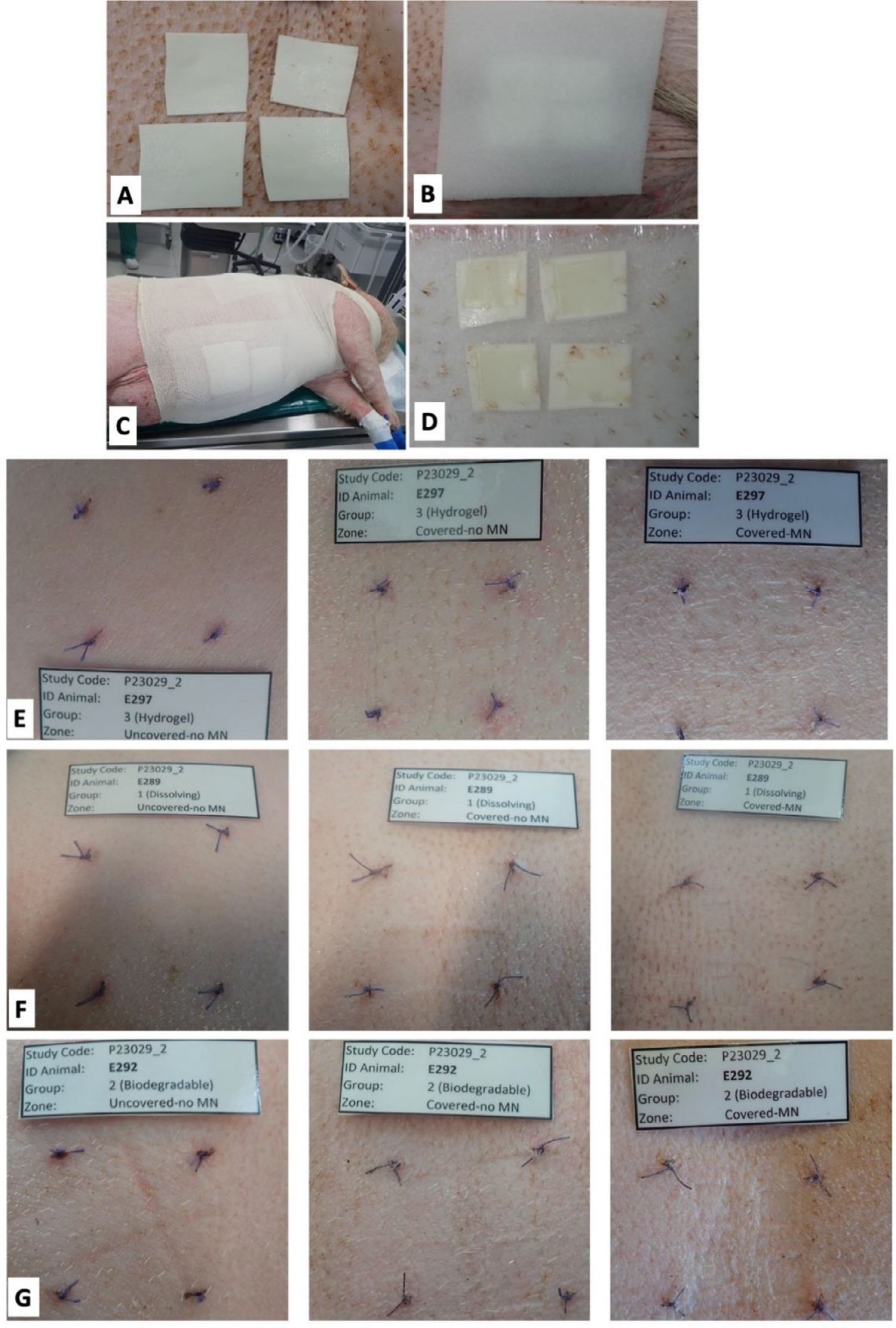
(A) MAPs supported by microfoam tape were applied to the miniature pig skin, while microfoam tape without MAPs was used for the control site. Both the MAP‐treated and control sites were covered with (B) an adhesive foam bandage, followed by (C) Omnifix elastic and a jacket‐type dressing made from an elastic tubular support bandage. (D) MAPs after removal from the skin. Representative images of skin from different sites under various conditions: covered with MAPs, covered without MAPs, and uncovered. These show no signs of erythema for the different MAP types: (E) hydrogel‐forming MAPs, (F) dissolving MAPs, and (G) implantable MAPs. Pictures were taken on day 7 of the study.

Erythema was assessed to evaluate the potential for skin irritation associated with repeated MAP applications over extended periods. The treated areas were monitored for erythema, swelling, and firmness. **Figure** **3**A–C illustrates the MAP application process, beginning with attaching the MAPs to the sticky side of a microfoam tape, followed by application to the skin. The application sites were then sequentially covered with an adhesive foam bandage, Omnifix elastic, and a jacket‐type dressing made from an elastic tubular support bandage placed around the animal's trunk to secure the MAPs during the study. Figure 3D shows the MAPs after removal from the skin.

Photographs of the treated areas were taken after MAP removal on each study day. No macroscopic reactions were observed in any animal throughout the study. Clinical images of skin treated with hydrogel‐forming MAPs, dissolving MAPs, and implantable MAPs are shown in Figure 3E–G, respectively. The total scores for erythema/inflammation, swelling, and firmness, assessed from medical photographs taken throughout the study, are detailed in **Table** **1**. All recorded scores for each parameter were 0, indicating no instances of erythema reaching moderate or severe levels, even after multiple MAP applications. These findings demonstrate the absence of skin irritation or adverse reactions following repeated MAP use.

**Table 1:** Evaluation of clinical photographs of miniature pigs following MAP application during the study.

	D0	D2	D7	D9	D14	D16	D21	D23	D28
Hydrogel‐forming MAPs	0	0	0	0	0	0	0	0	0

	D0	D2	D7	D9	D14	D16	D21	D23	D28
Dissolving MAPs	0	0	0	0	0	0	0	0	0
Implantable MAPs	0	0	0	0	0	0	0	0	0

These findings align with previously published studies evaluating the clinical application of MAPs. Al‐Kasasbeh *et al.* reported that repeated applications of hydrogel‐forming MAPs to human volunteers caused no erythema in most participants.^[^ ^20^ ^]^ While some volunteers exhibited mild erythema following hydrogel‐forming MAP application, this resolved shortly thereafter. Similar behavior was observed in related studies using a mouse model for hydrogel‐forming and dissolving MAPs.^[^ ^19^ ^]^ Additionally, a single application of implantable MAPs made from PLGA showed comparable results.^[^ ^49^ ^]^

Li *et al.* similarly reported that the application of dissolving MAPs to human volunteers was generally well tolerated, with no erythema in most cases.^[^ ^24^ ^]^ However, instances of moderate erythema were observed, particularly with MAPs featuring longer needles (1.2–1.5 mm). Finally, Hackethal *et al.* evaluated changes in microvasculature response to MAP application in human volunteers.^[^ ^25^ ^]^ They also reported a correlation between erythema formation and needle length. Moreover, they reported that the force used to apply the MAPs had an influence on the erythema. In all cases, the erythema disappeared between 12 and 48 h after insertion. It is noteworthy that previous studies primarily focused on the effects of single or repeated applications of one or two types of MAPs in either human or animal models. In contrast, the present study provides a comprehensive evaluation of the effects of repeated applications across the main types of MAPs described in the literature for drug delivery applications.

### 2.3 Measurement of Skin Integrity

The skin's water content follows a gradient, with higher levels in the deeper dermal layers that gradually decrease toward the surface, driving water diffusion from the dermis to the environment.^[^ ^50^ ^]^ The *stratum corneum* acts as the primary barrier to water and ion movement, making it a key parameter for assessing skin barrier integrity.^[^ ^51^ ^]^ Under normal conditions, transepidermal water loss (TEWL) reflects the natural movement of water between the skin and the external environment. An increase in TEWL indicates a compromised skin barrier, allowing excessive water loss from the dermis.^[^ ^52^ ^]^ Microneedle application creates temporary pores in the skin, leading to an initial increase in TEWL.^[^ ^51^ ^]^ TEWL is thus a crucial marker of skin barrier function, with elevated values signifying skin disruption. Previous studies have shown that these micropores close within 48–72 h under occlusive conditions.^[^ ^53^, ^54^ ^]^ In this study, TEWL was measured to evaluate skin barrier recovery and compare conditions before and after MAP application under three scenarios: skin sites covered with MAPs, sites covered without MAPs, and uncovered control sites.

**Figure 4:**
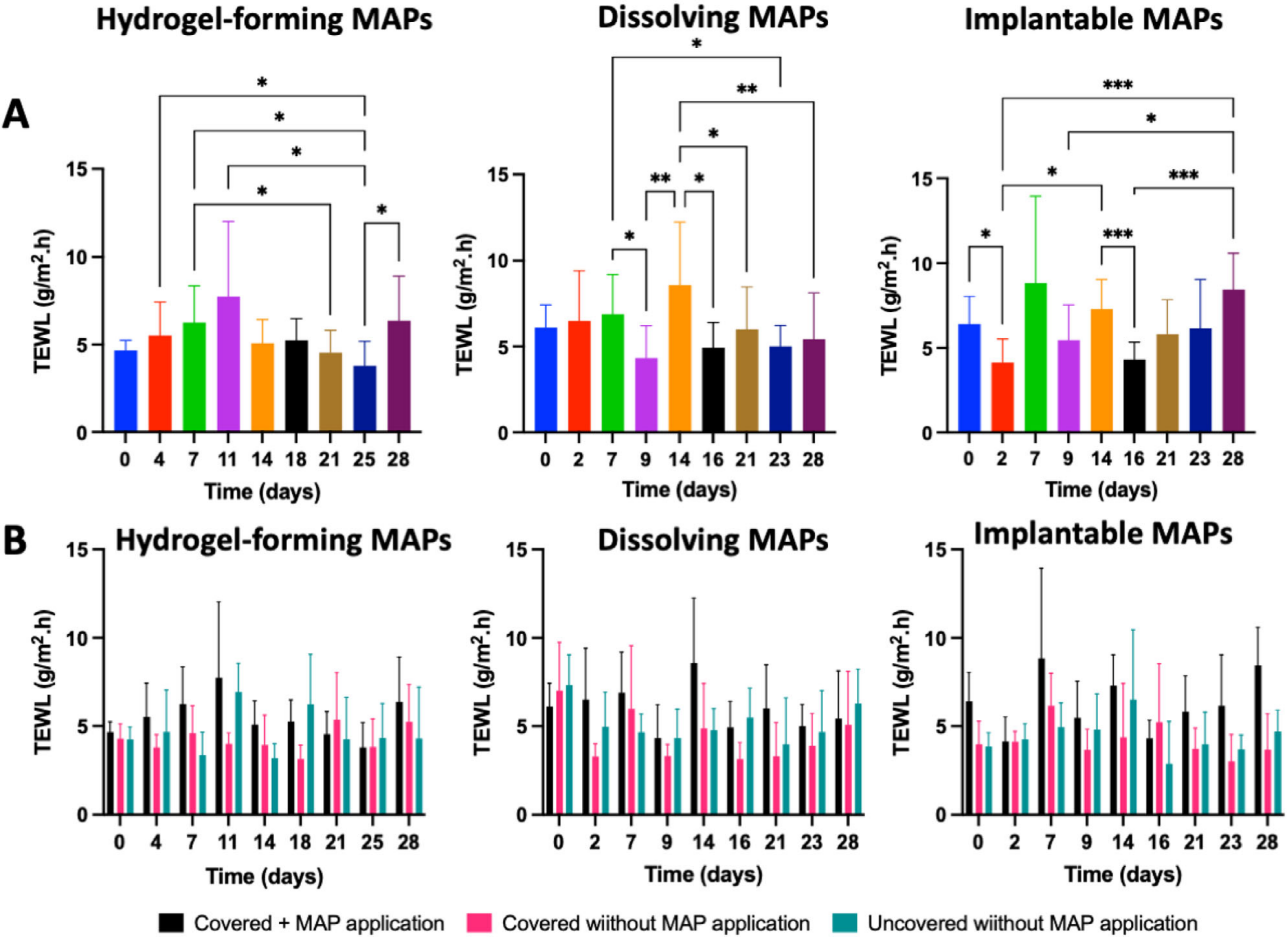
TEWL values were measured at predetermined time points during the study. (A) TEWL values at the MAP application site on miniature pig skin (means + SD, n = 12). (B) Comparison of TEWL values at different sites: MAP application site, covered skin without MAP, and uncovered skin (means + SD, n = 12).

**Figure** **4**A presents TEWL values at MAP application sites for hydrogel‐forming, dissolving, and implantable MAPs. On day 0, TEWL values were 6.12 ± 1.31 g m^−^^2^·h, 6.42 ± 1.63 g m^−^^2^·h, and 4.68 ± 0.56 g m^−^^2^·h for hydrogel‐forming, dissolving, and implantable MAPs, respectively. For hydrogel‐forming MAPs, TEWL values fluctuated during the study, as MAPs were applied for three days, removed, and replaced the same day over 28 days. Despite these fluctuations, TEWL values remained below 15 g m^−^^2^·h, aligning with control miniature pig skin values reported in prior in vivo studies.^[^ ^50^ ^]^ While significant differences in TEWL were observed on day 25 compared to days 4, 7, 11, and 28 (*p* < 0.05), these variations likely stemmed from minor differences in uncontrolled ambient conditions such as temperature, ventilation, and humidity.^[^ ^55^, ^56^ ^]^ Importantly, no significant difference was noted between day 0 and day 28 (*p* > 0.05), indicating that repeated MAP application did not compromise the skin barrier.

A similar trend was observed in the dissolving and implantable MAP groups. MAPs in these groups were applied on the first day of the week, removed after 48 h, and re‐applied after a four‐day “rest”. Although significant differences in TEWL values were detected on specific days (*p* < 0.05), no significant changes were observed between day 0 and day 28 (*p* > 0.05). These results suggest that repeated MAP application across all groups is safe and does not disrupt skin barrier function.

Figure 4B compares TEWL values at MAP application sites with control sites (skin covered without MAPs and uncovered skin). Significant differences in TEWL were observed between skin with MAPs and skin covered without MAPs, between skin with MAPs and uncovered skin, and between skin covered without MAPs and uncovered skin, as detailed in Table S1 (Supporting Information). However, despite these differences, TEWL values across all conditions remained below 10.5 g m^−^^2^·h. For context, non‐damaged skin typically exhibits TEWL values of ≈15 g m^−^^2^·h, while damaged skin, such as wounds, demonstrates TEWL values near 150 g m^−^^2^·h.^[^ ^18^ ^]^ In this study, all TEWL values were well below the threshold for damaged skin and consistent with previous findings for pig skin.^[^ ^50^ ^]^ These findings align with human studies, where repeated hydrogel‐forming MAP application did not compromise skin barrier integrity.^[^ ^20^ ^]^

The absence of significant differences in TEWL between time points and application sites highlights the rapid recovery of pores created by MAP insertion. This recovery reflects the skin's natural repair mechanisms, which involve lamellar body secretion and lipid synthesis to restore the *stratum corneum*.^[^ ^57^, ^58^ ^]^ Additionally, prostaglandins and thromboxane A2, key eicosanoids involved in inflammatory responses, play a crucial role in the wound‐healing process.^[^ ^59^ ^]^ Cyclooxygenase (COX) enzymes (COX‐1 and COX‐2) are responsible for converting arachidonic acid into prostaglandin endoperoxide, leading to the production of prostaglandins and thromboxane A2. In the skin, COX‐1 functions as a “housekeeping” enzyme involved in normal homeostasis, while COX‐2 is upregulated in response to skin injury. Nonsteroidal anti‐inflammatory drugs (NSAIDs) inhibit both COX enzymes, and studies in rat skin have shown that COX inhibition significantly delays wound healing, including the closure of pores created by microneedle insertions.^[^ ^51^, ^53^, ^59^, ^60^ ^]^

The duration of skin recovery depends on several factors, including MAP geometry, needle dimensions, and insertion duration.^[^ ^61^ ^]^ The findings of this study further support evidence of rapid skin barrier recovery following MAP removal, with TEWL values normalizing faster than anticipated. However, in future studies, TEWL measurements should be taken immediately after MAP removal to capture the temporary spike caused by open skin pores before full recovery. These results confirm that repeated MAP application over one month is safe and does not adversely affect skin integrity. The findings align with previous studies conducted in mouse models (dissolving and hydrogel‐forming MAPs) and human volunteers (hydrogel‐forming MAPs), where TEWL values returned to baseline after each MAP application, even following repeated use.^[^ ^19^, ^20^ ^]^

### 2.4 Skin Collection and Histopathology

**Figure 5:**
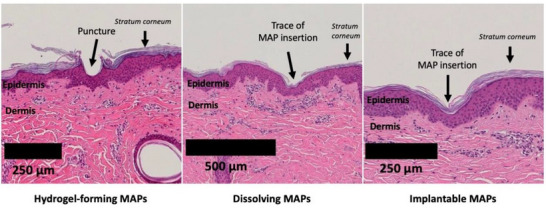
Histological analysis of miniature pig skin tissue stained with H&E, collected at the conclusion of the in vivo study (Day 28). The images illustrate skin tissue from different groups, including hydrogel‐forming MAPs, dissolving MAPs, and implantable MAPs, highlighting the structural effects of repeated MAP application.

To assess the effects of repeated MAP applications on skin integrity and the potential for inducing lesions in miniature pigs, skin samples were collected at the conclusion of the in vivo study (Day 28) and analyzed via light microscopy using hematoxylin and eosin (H&E) staining. The visualization of skin layers provided insights into the microstructural changes induced by MAPs, as shown in **Figure** **5**.

MAPs are designed to penetrate the *stratum corneum* and create microchannels that facilitate the transdermal delivery of active compounds. These microchannels disrupt the skin barrier while limiting their impact to the superficial dermis, enabling pain‐free drug delivery. Interestingly, the histological images revealed that the *stratum corneum* punctures were clearly visible in the group treated with hydrogel‐forming MAPs, while no punctures were discernible in the groups treated with dissolving MAPs or implantable MAPs. Instead, the skin in these groups showed signs of healing, with no residual punctures evident.

This discrepancy can be attributed to differences in the timeline of MAP application and removal. As detailed in Table 3, hydrogel‐forming MAPs were removed on Day 28, immediately prior to skin collection. In contrast, dissolving MAPs and implantable MAPs were removed on Day 23, and the skin was collected five days later. This additional time likely allowed the skin to undergo its natural repair mechanisms, resulting in the closure of microchannels. The rapid repair process is consistent with previous findings, where lamellar body secretion and lipid synthesis were initiated upon disruption of the *stratum corneum* to restore the barrier function.^[^ ^58^, ^62^ ^]^ In human studies, the skin barrier function has been shown to return to baseline levels within 18 h of hydrogel‐forming MAP removal.^[^ ^20^ ^]^

The histopathological analysis further demonstrated that repeated MAP application over a one‐month period did not induce significant lesions or adverse effects at the MAP application sites in any experimental group. Observed histological findings fell within the normal range of background lesions typically seen in miniature pigs of this strain and age under the study's conditions. This confirms that the repeated use of MAPs is safe and does not compromise skin integrity over extended periods.

### 2.5 Assessment of Clinical Signs

In this study, we assessed and monitored the clinical signs of miniature pigs to detect potential pain, distress, health changes, or any adverse effects associated with repeated MAP applications over a one‐month period. **Table** **2** outlines the detailed welfare scale, which was evaluated daily for 28 days. No animals exhibited significant changes in body condition, skin integrity, posture, general behavior, digestive or respiratory function, hydration status, motor activity, or nervous system function. Throughout the study, the animals maintained normal behavior, appetite, and posture. Additionally, no vocalizations, self‐mutilation behaviors, or signs of distress were observed, and no animals required euthanasia due to welfare concerns. Furthermore, no signs of infection were detected, as body temperature remained stable throughout the study. These findings confirm that repeated MAP applications were safe and did not negatively affect the health or welfare of the miniature pigs.

**Table 2:** The welfare scale was evaluated every day from D0 to D27.

Day	Hydrogel‐forming MAPs	Dissolving MAPs	Implantable MAPs	Day	Hydrogel‐forming MAPs	Dissolving MAPs	Implantable MAPs
D0	0	0	0	D14	0	0	0
D1	0	0	0	D15	0	0	0
D2	0	0	0	D16	0	0	0
D3	0	0	0	D17	0	0	0
D4	0	0	0	D18	0	0	0
D5	0	0	0	D19	0	0	0
D6	0	0	0	D20	0	0	0
D7	0	0	0	D21	0	0	0
D8	0	0	0	D22	0	0	0
D9	0	0	0	D23	0	0	0
D10	0	0	0	D24	0	0	0
D11	0	0	0	D25	0	0	0
D12	0	0	0	D26	0	0	0
D13	0	0	0	D27	0	0	0

These results are promising and align with previous studies evaluating infection risk and adverse events following MAP application. A study in mice reported no signs of infection post‐MAP application, and the animals remained healthy, continuing to gain weight throughout the study.^[^ ^19^ ^]^ Additionally, an in vitro study demonstrated that MAP punctures resulted in significantly lower microbial penetration compared to hypodermic needle punctures.^[^ ^63^ ^]^ Notably, no microorganisms crossed the viable epidermis in microneedle‐punctured skin, whereas microbial penetration was observed in hypodermic needle‐punctured skin.^[^ ^63^ ^]^ In vivo, these findings suggest that when MAPs are applied correctly, they are unlikely to cause local or systemic infections in immunocompetent individuals under normal circumstances.^[^ ^63^ ^]^

### 2.6 Urine Collection and Analysis

**Figure 6:**
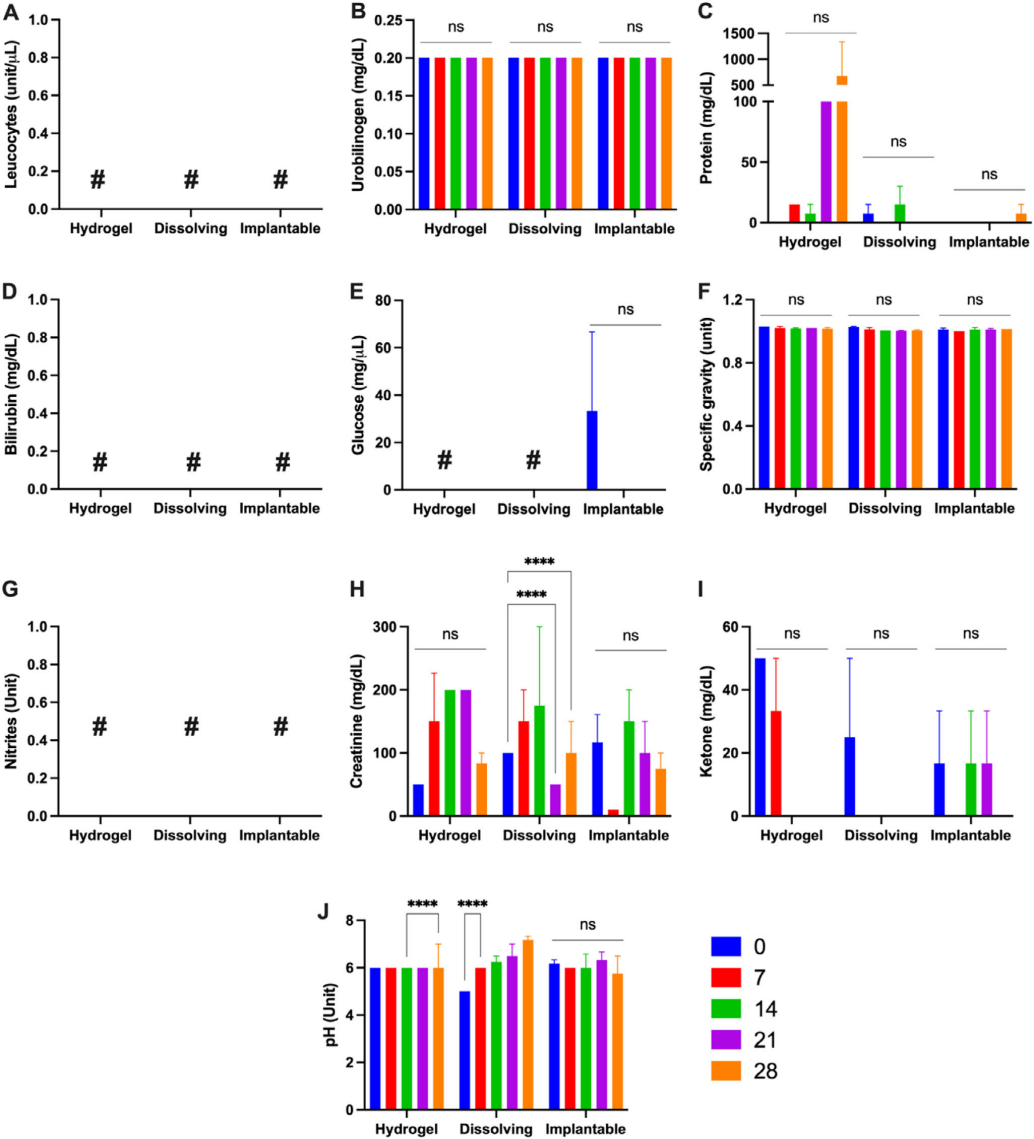
Weekly evaluation of urine parameters from miniature pigs treated with MAPs. Parameters assessed include (A) leukocytes, (B) urobilinogen, (C) protein, (D) bilirubin, (E) glucose, (F) specific gravity, (G) nitrites, (H) creatinine, (I) ketones, and (J) pH (means + SD, n = 3). The ‘#’ symbol indicates values below the lower limit of quantification (LLOQ) or undetected in the samples. Reference normal range of urinalysis using urine dipstick: leukocyte (negative), urobilinogen (<2.0 mg dL^−1^), protein (negative), bilirubin (negative), glucose (negative), specific gravity (1.003–1.030), Nitrite (negative), creatinine (20–400 mg dL^−1^), ketone (negative) and pH (5–9).

Urinalysis was performed weekly during the study to evaluate kidney toxicity and the systemic condition of the animals. Urinalysis assessed ten parameters: leukocytes, urobilinogen, protein, bilirubin, glucose, specific gravity, nitrites, creatinine, ketones, and pH. The results are summarised in **Figure** **6**.

No leukocytes, bilirubin, or nitrites were detected in the urine of any group throughout the study. The absence of leukocytes indicates no pyuria caused by infection, while the absence of bilirubin suggests healthy liver function.^[^ ^64^ ^]^ Similarly, the lack of nitrites confirms that the animals were free from bacterial urinary infections.^[^ ^65^ ^]^ Glucose was detected in the urine of one animal in the implantable MAP group on day 0, but was absent in subsequent weeks, indicating a transient metabolic condition unrelated to MAP application.^[^ ^66^ ^]^ A transient presence of glucose was observed in the urine of one animal in the implantable MAP group on day 0, which was not detected in any subsequent samples. This isolated finding is likely unrelated to MAP application and may reflect a temporary physiological fluctuation or stress‐related metabolic response. Importantly, glucose was undetectable in all other animals across all groups throughout the study, supporting the absence of any sustained glycaemic disturbance. Additionally, low levels of urobilinogen (0.2 mg dL^−1^) were consistently recorded in all animals. These levels fall below the normal detection range and are considered indicative of normal hepatic function.^[^ ^67^ ^]^

Protein excretion was detected in urine samples, with the highest value (2 mg) observed on day 28 in a single animal from the hydrogel‐forming MAP group. This level remains well below the clinical threshold for nephrotic‐range proteinuria (3.5 g day^−1^) ^[^ ^68^ ^]^ and is not considered indicative of renal impairment. In the dissolving and implantable MAP groups, protein levels consistently remained under 150 mg day^−1^, which is within the range of normal kidney function.^[^ ^68^ ^]^ These findings suggest no clinically relevant impact on renal physiology, and the isolated increase in one subject may reflect normal biological variation rather than a MAP‐related effect.

Specific gravity, an indicator of the kidney's ability to concentrate or dilute urine, ranged from 1.005 to 1.030 across all groups. This range suggests normal kidney function and adequate hydration in all animals.^[^ ^69^ ^]^ Creatinine levels were measured between 50 and 300 mg dL^−1^ across all groups, falling within the normal range and confirming that repeated MAP application over one month did not induce renal damage.^[^ ^70^ ^]^ Ketones were detected sporadically across the study period. On day 0, ketonuria was observed in all groups, with additional occurrences during the first week in the hydrogel‐forming MAP group and on days 14 and 21 in the implantable MAP group. With the exception of a single elevated value in the hydrogel‐forming group on day 0, all ketone levels remained below 40 mg dL^−1^, indicating moderate concentrations that are not typically associated with pathological conditions.^[^ ^71^ ^]^ These transient fluctuations are likely reflective of metabolic variability or dietary influences rather than MAP application. Urine pH varied between 5.0 and 8.0, remaining within the normal physiological range throughout the study.^[^ ^72^ ^]^ In conclusion, the urinalysis results indicate that repeated MAP application over one month did not cause significant renal damage or systemic adverse effects in miniature pigs. However, isolated findings, such as protein excretion and ketone presence, suggest the need for further evaluation in specific cases.

### 2.7 Haematological and Serum Biochemical Analysis

**Figure 7:**
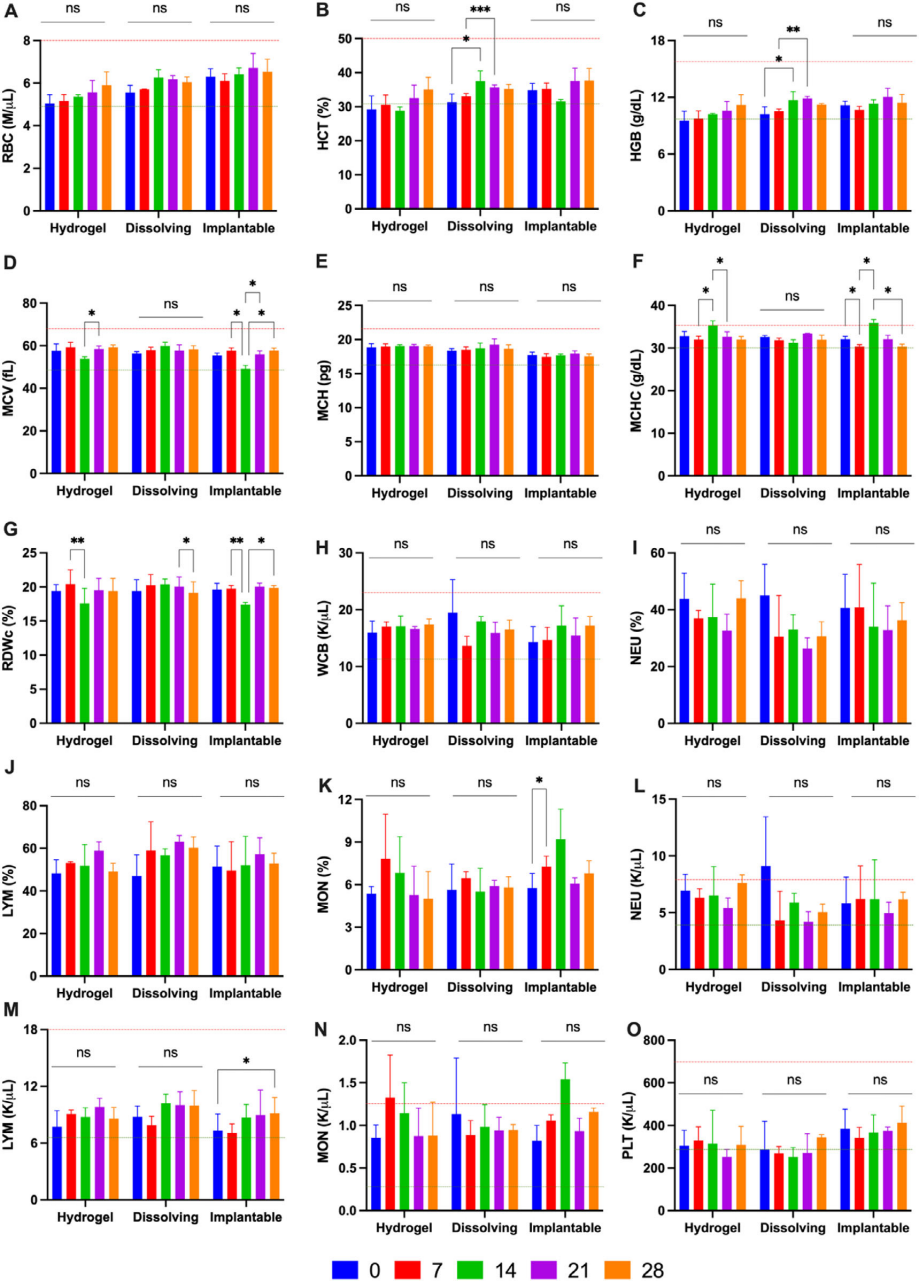
Weekly hematological evaluation of miniature pigs treated with MAPs. The parameters analyzed include (A) red blood cell (RBC) count, (B) hematocrit (HCT), (C) hemoglobin (HGB), (D) mean corpuscular volume (MCV), (E) mean corpuscular hemoglobin (MCH), (F) mean corpuscular hemoglobin concentration (MCHC), (G) red cell distribution width (RDWc), (H) white blood cell (WBC) count, (I,L) neutrophils (NEU), (J,M) lymphocytes (LYM), (K,N) monocytes (MON), and (O) platelets (PLT) (means + SD, n = 3). The red dashed line indicates the upper limit, while the green dashed line represents the lower limit of the reference range. Reference normal range of hematological evaluation: RBC (5.00–8.00 M µL^−1^), HCT (32.0–50.0%), HGB (10.7–16.7 g dL^−1^), MCV (50.0–68.0 fL), MCH (17.0–21.0 pg), MCHC (30.0–34.0 g dL^−1^), WCB (11.00–22.00 K µL^−1^), NEU (4.48–7.52 K µL^−1^), LYM (6.60–18.70 K µL^−1^), MON (0.30–1.25 K µL^−1^), PLT (300–700 K µL^−1^).

Haematological biomarkers in blood and serum are critical indicators of systemic health, enabling the assessment of potential infections, irritation, or abnormalities resulting from the repeated application of hydrogel‐forming, dissolving, or implantable MAPs. These parameters also provide insights into the functioning of the peripheral immune system and signs of inflammation. As shown in **Figure** **7**, apart from neutrophils and monocytes, all assessed biomarkers remained within the normal range, with no significant differences observed between day 0 and the weekly evaluations (*p* > 0.05). Notably, neutrophil levels were initially above the normal limit on day 0, but decreased to within the normal range in subsequent weeks. Similarly, monocyte levels were elevated above the normal limit during the first two weeks of the study but normalized in the latter half. These findings suggest that repeated MAP application over 28 days did not cause significant changes in hematological parameters or adverse systemic effects in the miniature pigs.

**Figure 8:**
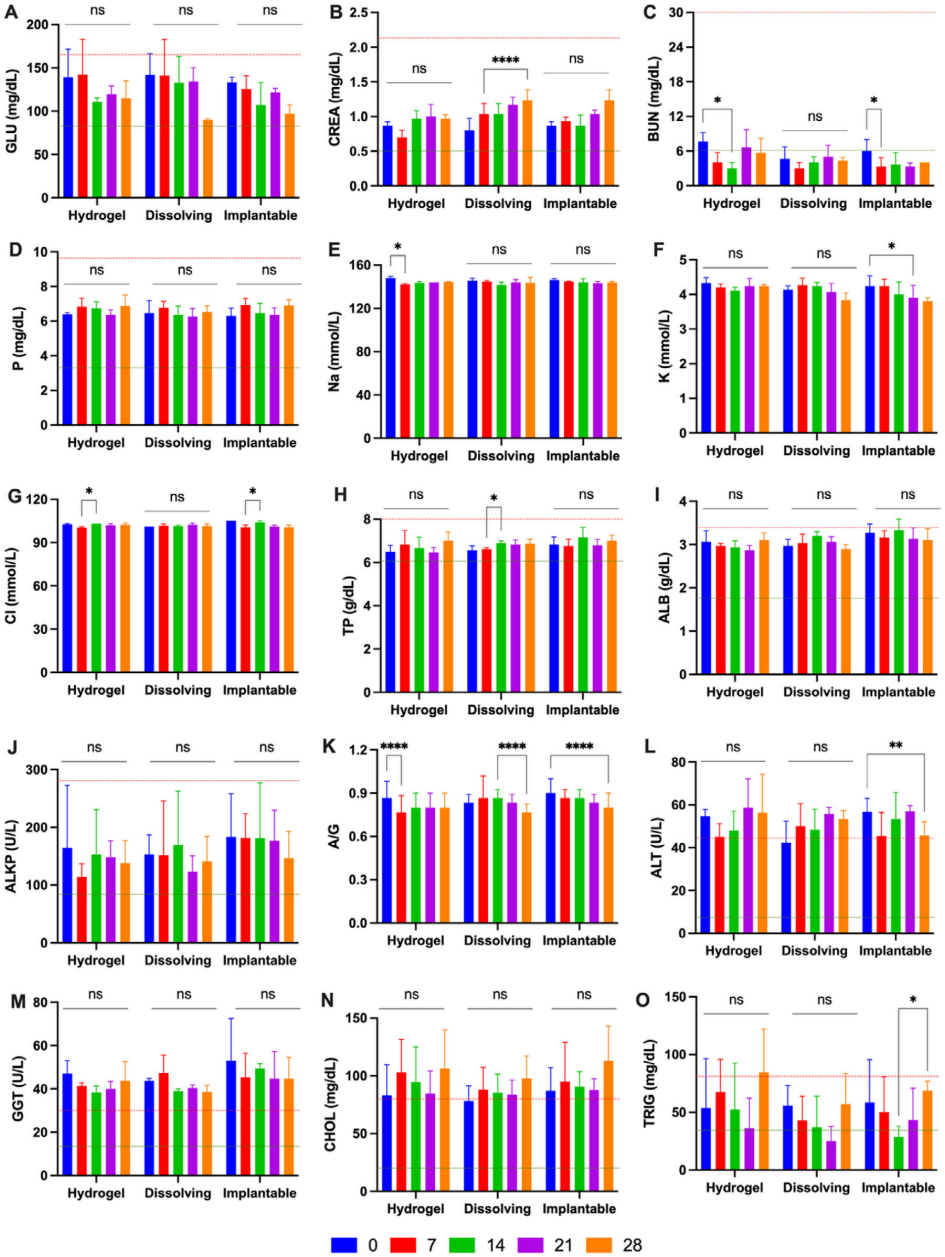
Weekly serum biochemical evaluation of miniature pigs treated with MAPs. The parameters analyzed include (A) glucose (GLU), (B) creatinine (CREA), (C) urea (BUN), (D) phosphorus (P), (E) sodium (Na), (F) potassium (K), (G) chloride (Cl), (H) total protein (TP), (I) albumin (ALB), (J) alkaline phosphatase (ALKP), (K) albumin‐to‐globulin ratio (A/G), (L) alanine aminotransferase (ALT), (M) gamma‐glutamyltransferase (GGT), (N) cholesterol (CHOL), and (O) triglycerides (TRIG) (means + SD, n = 3). The red dashed line indicates the upper limit, while the green dashed line represents the lower limit of the reference range. Reference normal range of biochemical evaluation: GLU (85–160 mg dL^−1^), CREA (0.5–2.1 mg dL^−1^), BUN (6–30 mg dL^−1^), P (3.6–9.2 mg dL^−1^), TP (6.0–8.0 g dL^−1^), ALB (1.8–3.3 g dL^−1^), ALKP (92–294 U L^−1^), ALT (9–43 U L^−1^), GGT (16–30 U L^−1^), CHOL 18–79 mg dL^−1^), TRIG (41–83 mg dL^−^).

Regarding biochemical evaluations (**Figure** **8**), most biomarkers, except for alanine aminotransferase (ALT), gamma‐glutamyltransferase (GGT), and cholesterol, were within the normal range, and no significant differences were noted between day 0 and the weekly assessments (*p* > 0.05). Although some parameters fell outside the reference ranges, these deviations were deemed clinically insignificant by the attending veterinarian. Overall, no relevant abnormalities or adverse effects were observed in the hematological or biochemical results, further confirming the systemic safety of repeated MAP application in miniature pigs.

### 2.8 ELISA Protocols for Biomarker Quantification in Plasma Samples

**Figure 9:**
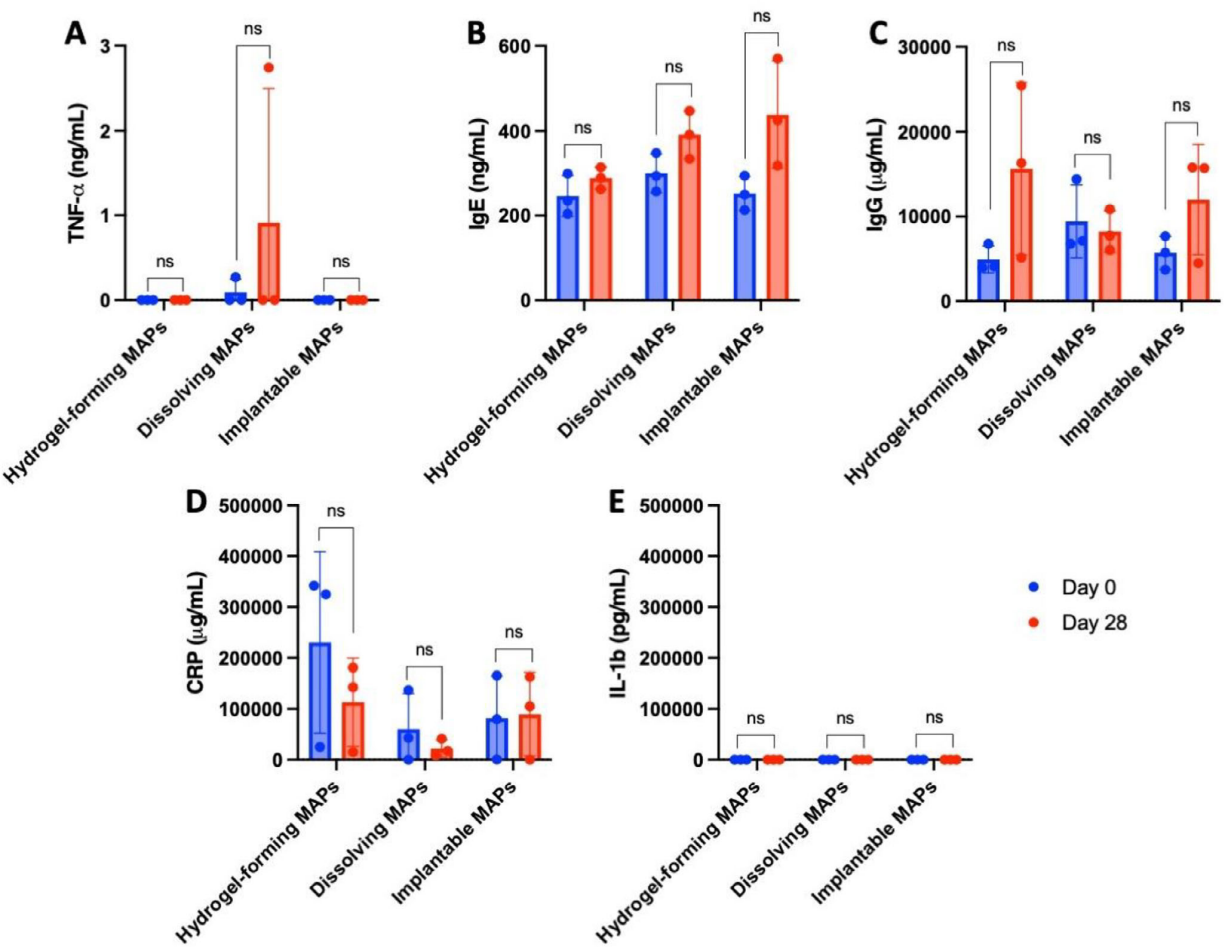
Plasma levels of (A) TNF‐α, (B) IgE, (C) IgG, (D) CRP, and (E) IL‐1β measured at the beginning (Day 0) and the end (Day 28) of the study using ELISA (means ± SD, n  =  3).

To investigate systemic biomarker responses following repeated MAP applications over a one‐month period, five distinct cytokines were quantified using ELISA kits. Plasma was separated from blood samples, and the concentrations of biomarkers on the final day of the study (Day 28) were compared to baseline levels (Day 0, prior to MAP insertion). Calibration curves were constructed in accordance with the manufacturers’ protocols to determine cytokine levels in the plasma samples. Tumor necrosis factor‐alpha (TNF‐α) levels are presented in **Figure** **9**A. No significant differences were observed between Day 0 and Day 28 for any MAP type (*p* > 0.05). Additionally, for hydrogel‐forming and implantable MAPs, all TNF‐α samples were below the detection limit. TNF‐α is a key inflammatory biomarker that modulates T‐cell proliferation and enhances resistance against various pathogens, including fungi, bacteria, and parasites.^[^ ^73^ ^]^ These results are consistent with a previous study that evaluated TNF‐α levels in a mouse model following repeated applications of dissolving and hydrogel‐forming MAPs. The polymers used in that study included PVP, Gantrez S‐97, and Gantrez‐based hydrogels.^[^ ^19^ ^]^ However, the study did not report results for implantable MAPs. In a separate study, hydrogel‐forming MAPs were tested in human volunteers, showing similar outcomes in terms of TNF‐α levels.^[^ ^20^ ^]^ Immunoglobulin E (IgE) levels were also assessed here, as IgE is a critical mediator of type‐1 hypersensitivity reactions, including allergic asthma, allergic rhinitis, food allergies, atopic dermatitis, drug allergies, and insect sting allergies.^[^ ^74^ ^]^ Elevated IgE levels can initiate a cascade of events leading to allergic contact urticaria, characterized by transient localized redness and swelling following direct exposure to an allergen.^[^ ^75^ ^]^ Figure 9B shows that IgE levels in plasma were not significantly different between Day 0 and Day 28 (*p* > 0.05). This finding indicates that repeated MAP application over one month did not trigger allergic reactions in any of the miniature pigs. Immunoglobulin G (IgG), the most abundant immunoglobulin in human plasma, constitutes 10–20% of plasma proteins and ≈75% of total immunoglobulins in healthy individuals.^[^ ^76^ ^]^ IgG antibodies are known for their high affinity and long persistence in circulation.^[^ ^78^ ^]^ Figure 9C demonstrates that IgG levels remained consistent between Day 0 and Day 28 across all MAP types (*p* > 0.05). This indicates that repeated applications of hydrogel‐forming, dissolving, and implantable MAPs did not induce allergic responses.

C‐reactive protein (CRP), a plasma protein produced by the liver, is widely used to diagnose infection and sepsis.^[^ ^78^, ^79^ ^]^ It is released into the bloodstream to activate the complement system, with levels rising rapidly within h of tissue injury or infection, thereby contributing to host defence.^[^ ^80^ ^]^ Due to its rapid response, short half‐life, and ease of measurement, CRP is a well‐established marker for detecting and monitoring systemic inflammatory responses.^[^ ^81^ ^]^ It remains stable in serum or plasma and is commonly used for infection and sepsis diagnosis.^[^ ^82^ ^]^ As shown in Figure 9D, CRP levels on day 28 were not significantly different from baseline (*p* > 0.05). This indicates the absence of infection throughout the study, confirming that repeated MAP applications did not induce systemic or skin infections. These findings are consistent with previous studies using similar MAP types in different models,^[^ ^19^, ^20^ ^]^ where no significant increase in CRP levels was observed following repeated MAP application (*p* > 0.05).

Interleukin‐1 beta (IL‐1β), a key cytokine in the interleukin family, plays a crucial role in regulating inflammation triggered by bacterial and viral infections.^[^ ^83^ ^]^ While primarily produced by innate immune cells, IL‐1β can also be expressed by certain skin epithelial cells, particularly in psoriatic conditions.^[^ ^84^ ^]^ Figure 9E shows that IL‐1β levels in plasma samples from all MAP groups (hydrogel‐forming, dissolving, and implantable MAPs) remained below the detection limits of the assay, indicating that IL‐1β was undetectable in the miniature pigs throughout the study. Collectively, these results suggest that repeated MAP applications over one month did not induce significant systemic immune responses, allergic reactions, infections, or inflammatory markers in the miniature pigs.

## 3 Conclusion

The safety of repeated MAP application is a crucial factor in their development as a transformative drug delivery platform. Unlike vaccines, MAPs for drug delivery require repeated applications, necessitating evaluation of their long‐term safety in a GLP industry‐standard model. Using miniature pigs as a translational model, this study provides robust evidence supporting the safety of hydrogel‐forming, dissolving, and implantable MAPs over a four‐week period. These findings are significant, as the skin structure and physiological responses of miniature pigs closely resemble those of humans, ensuring the clinical relevance of the results. Repeated MAP application did not compromise skin barrier function, as confirmed by TEWL measurements, which remained within normal ranges throughout the study. No skin reactions or adverse effects were observed at treated sites, as indicated by the modified Draize test values. Control areas, whether covered without MAPs or left uncovered, also showed no reactions, demonstrating the biocompatibility of both the MAP PB and dressing conditions. Systemic safety was thoroughly evaluated, with no significant differences in systemic immune responses, allergic reactions, infections, or inflammatory markers, including TNF‐α, IgE, IgG, CRP, and IL‐1β, between Day 0 and Day 28. This confirms that none of the tested MAPs triggered systemic immune activation, allergic responses, or inflammation. Similarly, hematological and biochemical analyses revealed no clinically relevant abnormalities, and urinalysis confirmed the absence of kidney toxicity or systemic effects. No weight loss or signs of infection were observed in the animals. Histopathological examination further validated the safety profile, revealing no significant lesions or adverse effects at application sites. Collectively, these findings demonstrate that hydrogel‐forming, dissolving, and implantable MAPs can be safely applied repeatedly without compromising skin integrity, inducing systemic inflammation, or causing other adverse effects. This study represents the first comprehensive GLP industry‐standard evaluation of all three leading MAP types for drug delivery. Previous studies either focused on a single MAP type, assessed only a single application or were not conducted under GLP conditions. The results support the continued development of MAPs as a safe and effective drug delivery platform, paving the way for broader clinical adoption. Additionally, this study contributes to establishing regulatory benchmarks for the preclinical evaluation of MAPs, facilitating their translation from animal studies to human clinical trials and eventual commercialization.

## 4 Experimental Section

### Preparation of Polymeric MAPs

**Figure 10:**
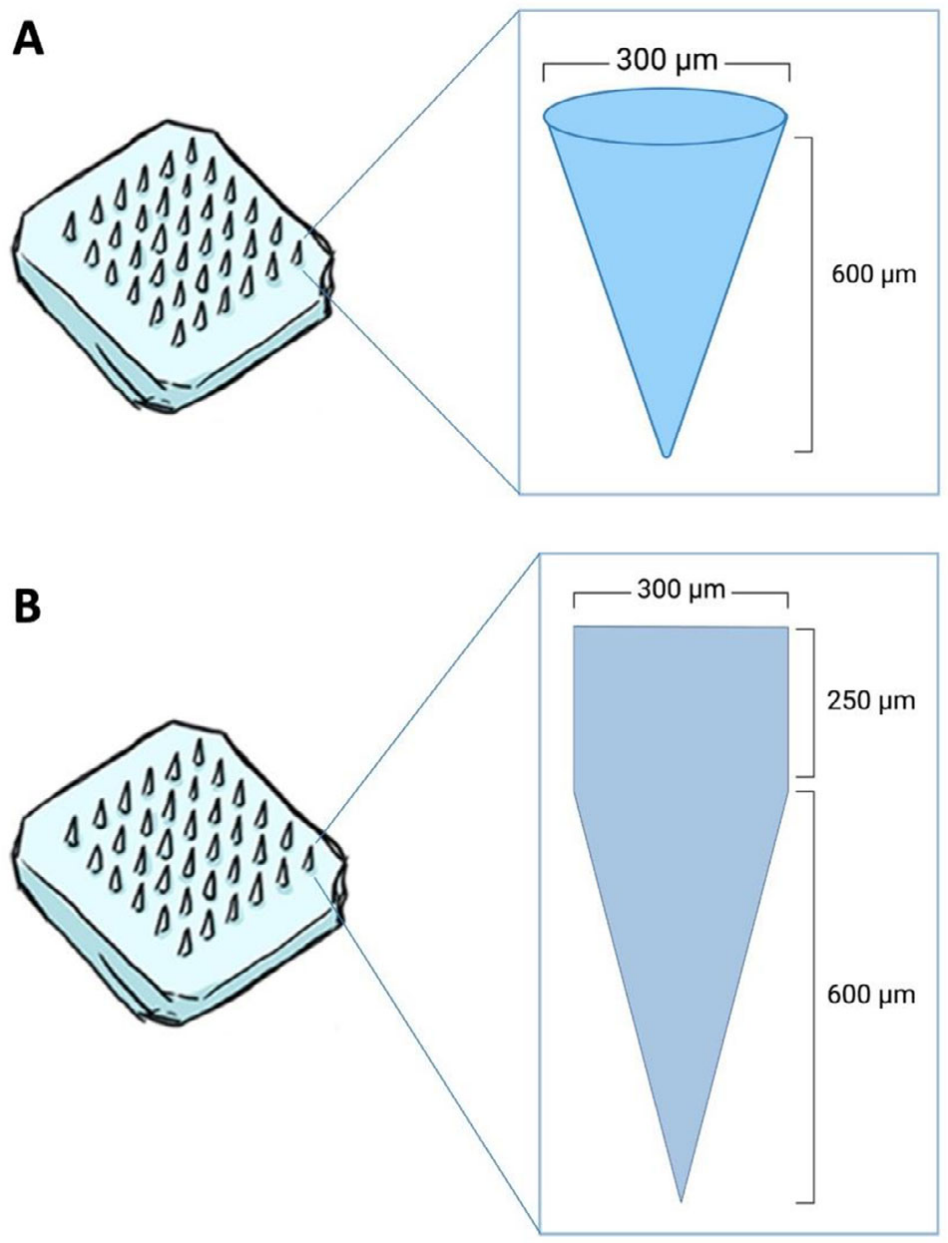
Illustration of needle size and geometry on the array of (A) hydrogel‐forming MAP, (B) dissolving and implantable MAPs.

**Figure 11:**
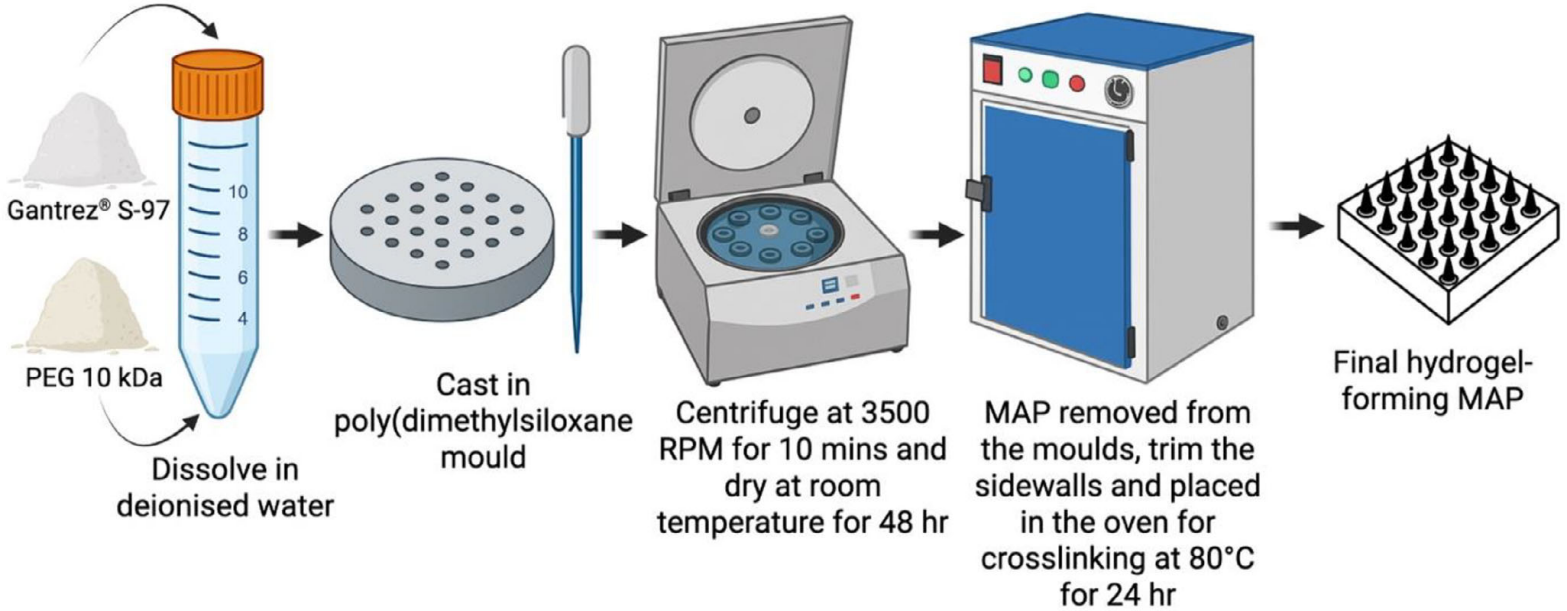
Schematic diagram illustrating the preparation process of hydrogel‐forming MAPs.

To prepare hydrogel‐forming MAPs, the required mass of Gantrez S‐97 (40% w/w stock solution) was weighed into a 50 mL Falcon tube. The stock solution was pre‐prepared by dissolving Gantrez S‐97 powder in deionized water and thoroughly mixing. Separately, PEG 10,000 was weighed into a 15 mL Falcon tube and dissolved in a minimal volume of deionized water. The PEG solution was then added to the Gantrez S‐97 solution, and the mixture was adjusted to the desired weight with deionized water. The blend was mixed with a spatula until homogeneous and centrifuged at 3500 RPM for 10 min to remove air bubbles. The formulation was cast into 11 × 11 pre‐formed silicone microneedle molds (conical shape, 600 µm height, 300 µm base width, and 300 µm interspacing, illustrated in **Figure** **10**A). As illustrated in **Figure** **11**, the moulds containing formulation were centrifuged at 3500 RPM for 10 min left to dry at room temperature for 48 h. After drying, sidewalls formed during the process were trimmed using scissors, and the MAPs were crosslinked in a hot air oven at 80°C for 24 h to create insoluble hydrogel‐forming structures.^[^ ^13^, ^27^ ^]^

**Figure 12:**
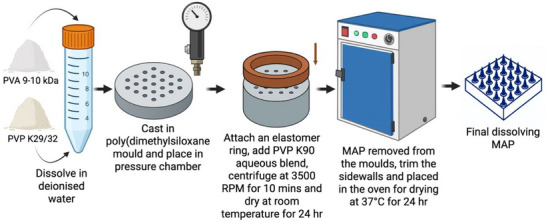
Schematic diagram illustrating the preparation process of dissolving MAPs.

For dissolving MAPs, separate aqueous stocks of PVA (9–10 kDa) and PVP (58 kDa) were prepared in deionized water at a polymer concentration of 40% w/w each. Equal volumes of the stocks were combined to create a blend with a final polymer concentration of 20% w/w. The mixture was briefly hand‐mixed, centrifuged to remove air bubbles, and inspected to ensure no phase separation occurred. The polymer blend was poured into a poly(dimethylsiloxane) mold containing 16 × 16 pyramidal‐cuboidal needles with dimensions of 850 µm height, 300 µm base width, and 300 µm interspacing, covering a patch area of 0.36 cm^2^, illustrated in Figure 10B. As displayed in **Figure** **12**, the molds were placed in a positive pressure chamber at 4 bar for 5 min. Excess formulation was carefully removed with a spatula and the molds were returned to the chamber for an additional 30 min under the same pressure. Elastomer rings (external diameter: 23 mm, internal diameter: 18 mm, thickness: 3 mm) were affixed on top of the molds using a glue solution prepared from an aqueous blend of 40% w/w PVA (9–10 kDa). The molds were allowed to dry at room temperature for 6 h. Subsequently, 850 µL of a second layer consisting of an aqueous blend of 30% w/w PVP (90 kDa) and 1.5% w/w glycerol was added to the molds. After centrifuging at 3500 RPM for 10 min, the molds were dried at room temperature for 24 h. Any sidewalls formed during the drying process were trimmed using scissors, and the molds were further dried at 37°C for 24 h.^[^ ^35^, ^42^, ^43^ ^]^

**Figure 13:**
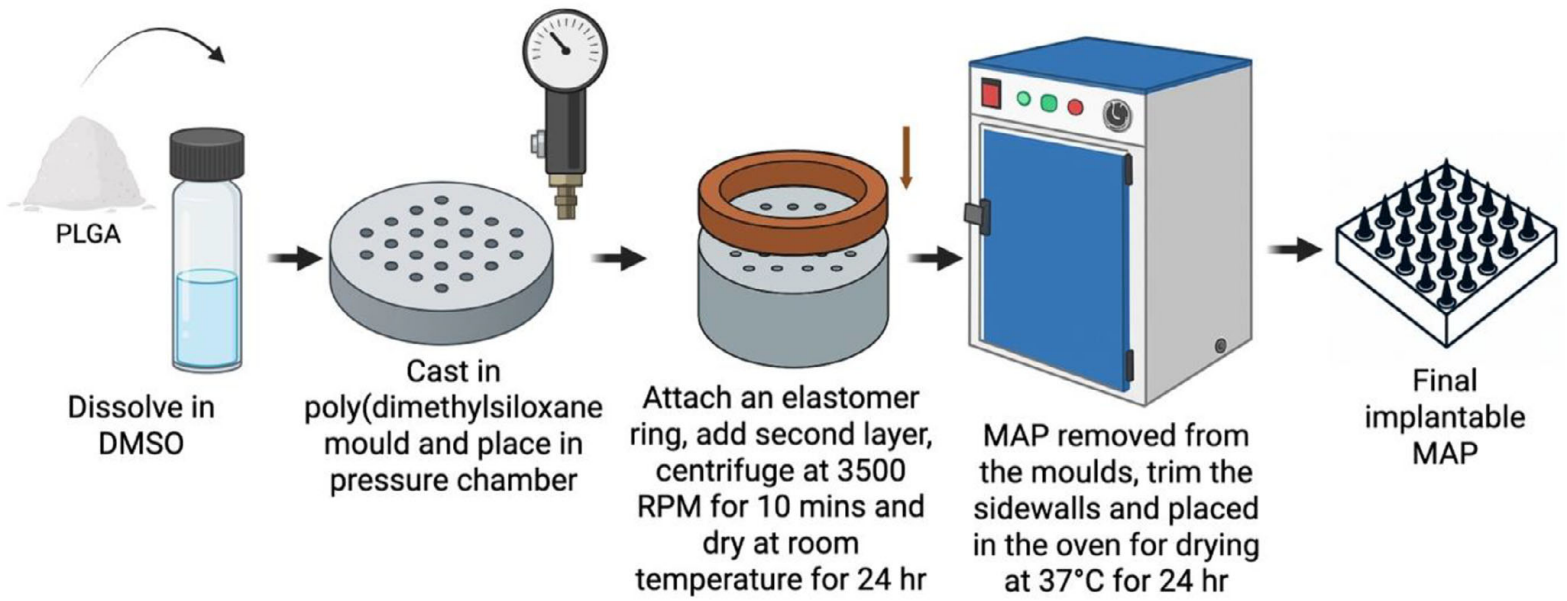
Schematic diagram illustrating the preparation process of implantable MAPs.

For implantable MAPs, 0.1 g of PLGA (10% w/v) was dissolved in 1 mL of DMSO using a SpeedMixer™ DAC 150.1 FVZ‐K (GermanEngineering, Hauschild & Co. KG, Hamm, Germany) for 3 min at 3000 RPM. The PLGA solution was then added to a poly(dimethylsiloxane) mold with 16 × 16 pyramidal‐cuboidal needles (the same geometry to dissolving MAP, illustrated in Figure 10B). The molds were placed in a positive pressure chamber at 4 bar for 5 min (**Figure** **13**). The excess formulation was scraped off with a spatula, and the molds were subjected to an additional 30 min of positive pressure under the same conditions. Elastomer rings (external diameter: 23 mm, internal diameter: 18 mm, thickness: 3 mm) were glued on top of the molds using a 40% w/w PVA (9–10 kDa) aqueous blend. After drying at room temperature for 6 h, 500 µL of a second layer (20% w/w PVA and PVP aqueous blend) was added to the molds. The molds were centrifuged at 3500 RPM for 10 min and dried at room temperature for 24 h. Sidewalls formed during drying were trimmed with scissors, and the molds were further dried at 37°C for 24 h.^[^ ^7^ ^]^

### Characterisation of Polymeric MAPs

The mechanical properties of the MAPs under compression were tested using a TA‐TX2 Texture Analyser (Stable Microsystems, Haslemere, UK) in compression mode, following previously established methods.^[^ ^30^, ^85^, ^86^, ^87^ ^]^ In brief, the arrays were compressed against an aluminum block applying 32 N of force for 30 s. The microneedle height was measured before and after testing using a digital light microscope (Leica EZ4 D, Leica Microsystems, Milton Keynes, UK). The insertion performance of the MAPs was evaluated with the same Texture analyzer and a validated skin model, Parafilm.^[^ ^48^, ^88^ ^]^ Eight layers of Parafilm were stacked to create a composite film ≈1 mm thick. MAPs were attached to a cuboidal probe (1 cm^2^ cross‐sectional area) with double‐sided adhesive tape, and the probe was moved downward at 1.19 mm s^−1^ until a force of 32 N was applied.^[^ ^88^ ^]^ This force was maintained for 30 s before the MAPs were removed. The Parafilm layers were then unfolded, and the number of holes created in each layer was counted using the light microscope. Additionally, MAP insertion into full‐thickness neonatal porcine skin was examined with an OCT microscope (EX1301 VivoSight, Michelson Diagnostics Ltd, Kent, UK). Needle insertion depths were measured using ImageJ software (National Institutes of Health, Bethesda, MD, USA).

### Animal and Species Selection

Miniature pigs (*Sus scrofa domesticus)* were selected for this study as they were widely recognized in translational dermatological research due to their anatomical and physiological similarities to humans.^[^ ^89^ ^]^ Their dermal tissue closely resembles human skin in terms of thickness, structure, and function, leading to comparable local and systemic exposure to tested compounds.^[^ ^90^ ^]^ Over recent decades, miniature pigs have been successfully utilized as a non‐rodent species in regulatory preclinical safety and tolerance studies for various drug classes, including steroids, vitamin D analogs, anti‐inflammatory medicines, and antibiotics.^[^ ^91^ ^]^ Additionally, their size allows for the administration of test volumes similar to those used in clinical trials.^[^ ^92^ ^]^

A total of nine healthy miniature pigs (six females and three males) were used in this study. Three animals (two females and one male) were assigned to each MAP type, namely hydrogel‐forming MAPs, dissolving MAPs, and implantable MAPs. All animals underwent a comprehensive clinical examination, blood analysis, and body weight measurement prior to the study to confirm their health status. The flanks were inspected to ensure the absence of scratches, wounds, or unresolved infections. A veterinarian compiled the findings from these evaluations into a document verifying that the animals met all inclusion criteria. Animal husbandry and daily monitoring were conducted. Veterinary care was available throughout the study, and animals were routinely examined by qualified study personnel for clinical signs or other health changes.

### Study Protocol

The study was approved by the ethical committee of Specific Pig (Specipig) S.L. and conducted in compliance with the OECD Principles of Good Laboratory Practice (revised in 1997, C (97) 186/Final, Paris, 26th November 1997), Directive 1999/11/CE (8th March 1999, EU), and Real Decreto 1369/2000 (19th July, Spain). The study was subject to periodic inspections, including facility and process‐based evaluations during its execution. The research was carried out at Specific Pig (Specipig) S.L. (Barcelona, Spain), a facility certified for GLP compliance in this type of research.

**Figure 14:**
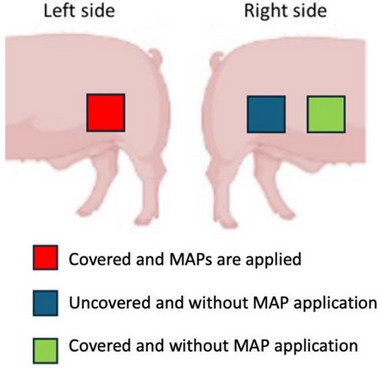
Representative image of the study areas.

**Figure 15:**
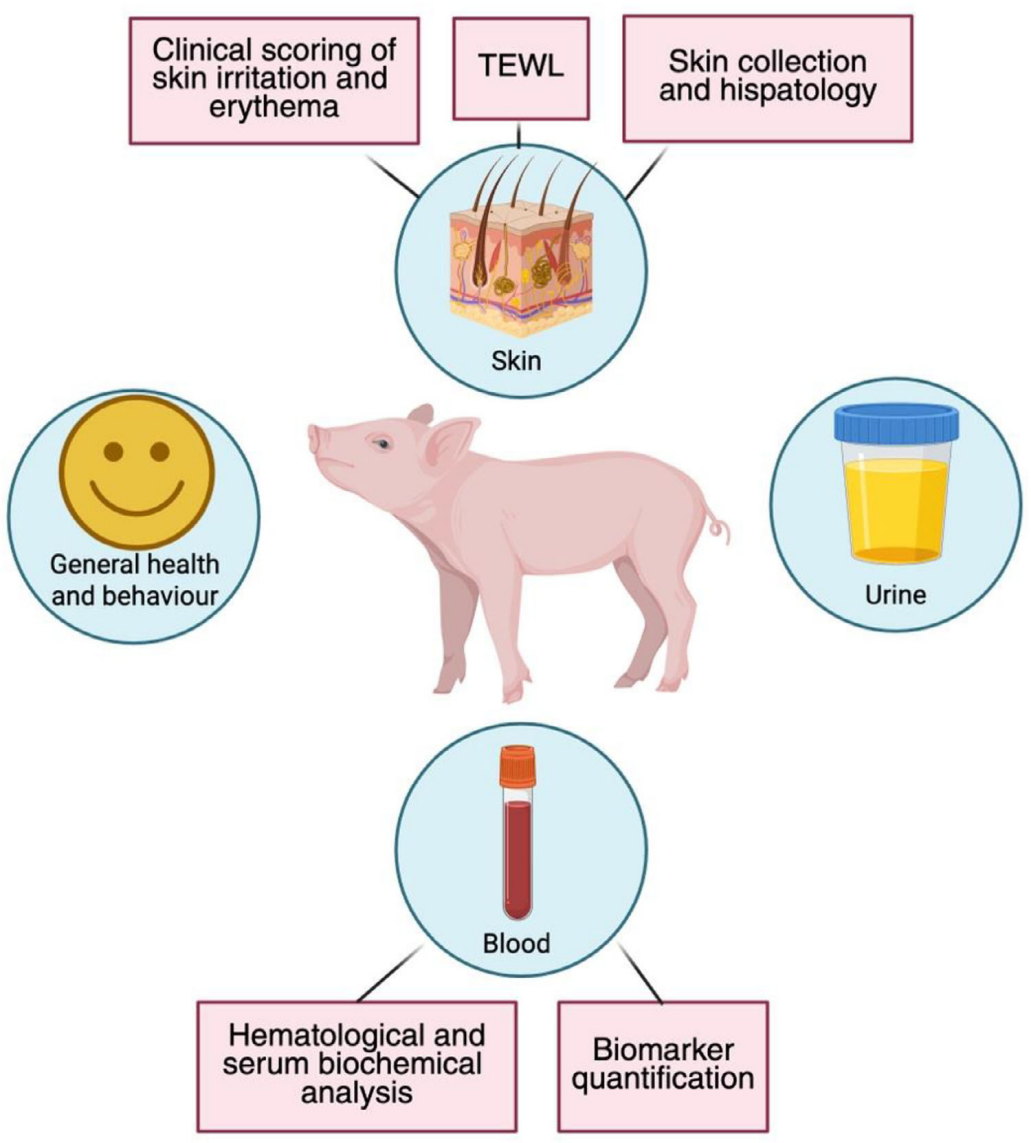
Schematic representation of the health assessment protocol and sample collection in minipigs following repeated application of MAPs over a 28‐day period.

As outlined in the study design (**Table** **3** and **Table** **4**), on designated application days, four MAPs of the same type were applied to the left flank of each animal, while the right flank served as a control. Two control areas were used: one covered with a dressing and another left uncovered, with neither receiving MAP application (**Figure** **14**). The study areas were marked using sutures at the four corners to ensure accurate placement of the MAPs. Throughout the procedure, animals were sedated with a combination of dexmedetomidine (0.03 mg kg^−1^), midazolam (0.3 mg kg^−1^), and butorphanol (0.3 mg kg^−1^). MAPs were applied to the caudal flank area (Figure 14), and treated regions were secured with Microfoam™ medical tape (3M™, Hutchinson, MN, USA), adhesive foam bandage (Snogg, Vennesla, Norway), and Omnifix elastic (Hartmann, Heidenheim, Germany). To maintain MAP placement throughout the study, a jacket‐type dressing made from an elastic tubular support bandage (Tubilast, Caribu Medical, Barcelona, Spain) was fitted around the animal's trunk. Health assessments and sample collections were conducted throughout the 28‐day period, following the repeated MAP application protocol, as illustrated in **Figure** **15**.

**Table 3:** The study design for the animal group received hydrogel‐forming MAPs.

Hydrogel‐forming MAPs				
Day ‐4	Day 0	Day 4	Day 7	Day 11
Trimming and cleaning the study areas Weight control and blood measurements	Trimming and cleaning the study areas Weight control and blood measurements MAP application TEWL, Draize Test, and temperature check Blood collection	MAP removal TEWL, Draize Test, and temperature check MAP application	MAP removal Weight control and blood measurements TEWL, Draize Test, and temperature check MAP application	MAP removal TEWL, Draize Test, and temperature check MAP application

Day 14	Day 18	Day 21	Day 25	Day 28
MAP removal Weight control and blood measurements TEWL, Draize Test, and temperature check MAP application	MAP removal TEWL, Draize Test, and temperature check MAP application	MAP removal Weight control and blood measurements TEWL, Draize Test, and temperature check MAP application	MAP removal TEWL, Draize Test, and temperature check MAP application	MAP removal Weight control and blood measurements TEWL, Draize Test, and temperature check Blood collection Necropsy

**Table 4:** The study design for the animal groups received dissolving MAPs and implantable MAPs.

Dissolving MAPs and implantable MAPs				
Day ‐4	Day 0	Day 2	Day 7	Day 9
Trimming and cleaning the study areas Weight control and blood measurements	Trimming and cleaning the study areas Weight control and blood measurements MAP application TEWL, Draize Test, and temperature check Blood collection	MAP removal TEWL, Draize Test, and temperature check	Trimming and cleaning the study areas Weight control and blood measurements TEWL, Draize Test, and temperature check MAP application	MAP removal TEWL, Draize Test, and temperature check

Day 14	Day 16	Day 21	Day 23	Day 28
Trimming and cleaning the study areas Weight control and blood measurements TEWL, Draize Test, and temperature check MAP application	MAP removal TEWL, Draize Test, and temperature check	Trimming and cleaning the study areas Weight control and blood measurements TEWL, Draize Test, and temperature check MAP application	MAP removal TEWL, Draize Test, and temperature check	Weight control and blood measurements TEWL, Draize Test, and temperature check Blood collection Necropsy

### Clinical Scoring of Skin Irritation and Erythema

Clinical scoring was employed to assess the presence of skin irritation, erythema, or other adverse reactions in the minipig study following MAP application. The condition of the treated regions was monitored both before and after patch removal to evaluate any changes over time. A detailed clinical scoring system for each test site is provided in **Table** **5**, allowing for standardized evaluation across all participants. Clinical photographs of the skin were captured before and after MAP application using controlled lighting conditions to ensure consistency and clarity. These images were later assessed blindly by an experienced veterinarian to minimize bias and ensure objective evaluation. The scoring focused on visible signs of irritation, erythema, and other skin reactions, with results contributing to the overall safety profile of the MAPs. This systematic approach provided a reliable method for documenting and analyzing the dermatological effects of MAP application.

**Table 5:** The grading scale for erythema/inflammation, swelling, and firmness parameters.

Scale	Erythema description
0	No erythema
1	Very slight erythema (barely perceptible)
2	Slight erythema
3	Moderate to severe erythema
4	Severe erythema (beet redness) to slight scar formation

Scale	Swelling description
0	No swelling
1	Very slight swelling
2	Slight swelling
3	Moderate swelling
4	Severe swelling

Scale	Firmness description
0	No perceptible difference in firmness after MAP application
1	Very slight firmness (barely perceptible)
2	Mildly firm
3	Moderately firm
4	Very firm

### Measurement of Skin Integrity

TEWL was measured using the VapoMeter (Delfin Technologies Ltd., Kuopio, Finland), a device equipped with a closed cylindrical chamber containing sensors for humidity and temperature. TEWL was calculated based on the linear increase in humidity within the chamber after the device was placed in contact with the skin. The evaporation rate was determined following Fick's Law of diffusion, representing the quantity transported per defined area and time. Results were reported in grams per square meter per hour (g/m^2^·h).

For hydrogel‐forming MAPs, TEWL measurements were conducted on D0 (before patch placement) and twice weekly after patch removal and before MAP re‐application. For dissolving and implantable MAPs, TEWL measurements were performed on D0 (before patch placement) and twice weekly after patch removal. Prior to measurement, the skin was cleaned using soft, semi‐wet tissue paper and allowed to air‐dry for 3 to 7 min. Four TEWL measurements were taken from each MAP application region, as well as from the control areas (uncovered without MAPs and covered without MAPs).

### Skin Collections and Histopathology

At the conclusion of the study (Day 28), the animals were euthanized, and a necropsy was conducted to collect skin samples from each study area, including regions covered with MAP applications, covered without MAP applications, and uncovered without MAP applications. Tissue samples were processed for histological analysis using hematoxylin and eosin staining. Light microscopy was performed by Patconsult LAB. S.L. to evaluate tissue architecture and identify any pathological changes potentially induced by MAP application. All stained slides were digitized using the NanoZoomer S10 digital slide scanner (Hamamatsu Photonics, Cerdanyola, Spain) and analyzed with NDP scan software (PAT14).

### Assessment of Clinical Signs

Clinical signs were assessed during each patch change under sedation. The examination included a comprehensive assessment of the animal's general health and behavior, including body condition, skin and fur quality, posture, motor activity, hydration status, and any signs of nervous system abnormalities such as tremors or paralysis. Specific systems evaluated included the digestive and respiratory systems. Additionally, body temperature was measured and recorded as part of the clinical assessment to monitor any deviations that could indicate systemic reactions.

### Urine Collection and Analysis

Urine samples were collected weekly using the cystocentesis method under sedation, with a target volume of 2 ± 1 mL per collection. Urine analysis was performed using urinalysis reagent strips (AccuDoctor, China) to assess multiple indicators of health. The parameters analyzed included leukocytes, urobilinogen, protein, bilirubin, glucose, ascorbic acid, specific gravity, ketones, nitrites, creatinine, pH, and blood. A color guide provided with the reagent strips was used for result interpretation. These measurements provided a comprehensive overview of renal function and systemic health throughout the study.

### Blood Collection

Blood samples were collected from the jugular veins of miniature pigs using 20G needles and EDTA‐containing Vacutainer tubes (Becton Dickinson, Swindon, UK). ≈10 mL of blood was withdrawn per collection. Before the procedure, the puncture site was cleaned with an alcohol swab and, after sampling, a sticking plaster (Band‐Aid™, Johnson & Johnson, Maidenhead, UK) was applied to minimize bleeding and maintain hygiene. Blood samples were collected at two‐time points: at the start of the study (day 0) and at the end (day 28). The collected blood samples were stored at 2–8°C until processing. Plasma was separated by centrifuging the blood at 2000 RPM for 15 min at 4°C using a Sigma 2–16k laboratory centrifuge (Sigma, Hamburg, Germany). The separated plasma was immediately transferred into pre‐sterilized Eppendorf tubes and analyzed within one hour of extraction. These assessments aimed to evaluate potential systemic effects or stress caused by repeated MAP applications. Changes in blood markers throughout the study were monitored as indicators of physiological responses to the treatment.

### Hematological and Serum Biochemical Analysis

Blood and serum samples were collected and analyzed using specialized equipment, with the operation supported by the Procyte Dx reagent kit and Procyte Dx dye kit (IDEXX Laboratories, Inc., Westbrook, ME, USA). Hematological parameters evaluated included red blood cell (RBC) count, hematocrit (HCT), hemoglobin (HGB), mean corpuscular volume (MCV), mean corpuscular hemoglobin (MCH), mean corpuscular hemoglobin concentration (MCHC), red cell distribution width (RDWc), white blood cell (WBC) count, neutrophils (NEU), lymphocytes (LYM), monocytes (MON), and platelets (PLT). For serum biochemical analysis, the parameters measured were glucose (GLU), creatinine (CREA), urea (BUN), phosphorus (P), sodium (Na), potassium (K), chloride (Cl), total protein (TP), albumin (ALB), alkaline phosphatase (ALKP), alanine aminotransferase (ALT), gamma‐glutamyltransferase (GGT), cholesterol (CHOL), and triglycerides (TRIG).

### Biomarker Quantification in Plasma Samples

Systemic responses to repeated MAP applications were assessed by quantifying key inflammatory and immunological biomarkers in plasma samples. Blood samples (2 ± 1 mL) were collected in sodium citrate tubes at specified intervals and stored at −20°C until analysis. Biomarkers measured included C‐reactive protein (CRP), interleukin‐1β (IL‐1β), tumor necrosis factor‐α (TNF‐α), immunoglobulin G (IgG), and immunoglobulin E (IgE). Commercial enzyme‐linked immunosorbent assay (ELISA) kits (MyBioSource, Inc., San Diego, CA, USA) were used according to the manufacturer's protocols.

Plasma samples were diluted as per the kits’ specifications: CRP (1:500), total IgG (1:500,000), IgE (1:10), IL‐1β (1:2), and TNF‐α (1:2). ELISA procedures involved adding samples and standard to pre‐washed 96‐well plates, incubating at room temperature or 37°C for 1–2 h, and washing to remove unbound antigens. Detection antibodies were applied, followed by incubation with streptavidin‐horseradish peroxidase (HRP). A colorimetric substrate was added, and the reaction was terminated with acid. Absorbance was measured at 450 nm using a BioTek Synergy HTX Fluorescence plate reader (Agilent, Santa Clara, CA, USA). These analyses provided insights into the potential systemic effects of the MAP treatments.

### Statistical Analysis

GraphPad Prism 10.4.1 was used for data entry and statistical analysis. One‐way analysis of variance (ANOVA) was used for comparisons when the data followed a normal distribution, while the Kruskal‐Wallis test was applied for non‐normally distributed data. Results were presented as mean values with standard deviations (SD). Statistical significance levels were indicated as follows: *p ≤ 0.05, **p ≤ 0.01, and ***p ≤ 0.001.

## Conflict of Interest

The authors declare no conflict of interest.

## Author Contributions

Q.K.A. performed in conceptualization, methodology, visualization, investigation, validation, formal analysis, data curation, project administration, resources, writing – original draft, writing – review and editing. A.R.J.H. performed in conceptualization, methodology. P.E.M. performed in conceptualization, resources, project administration. E.L. performed in conceptualization, methodology, project administration, supervision, writing – review and editing. R.F.D. performed in conceptualization, funding acquisition, project administration, resources, supervision, writing – review and editing.

## Supporting information



Supporting Information

## Data Availability

The data that support the findings of this study are available from the corresponding author upon reasonable request.

## References

[1] E. McAlister , M. Kirkby , J. Domínguez‐Robles , A. J. Paredes , Q. K. Anjani , K. Moffatt , L. K. Vora , A. R. J. Hutton , P. E. McKenna , E. Larrañeta , R. F. Donnelly , Adv. Drug Delivery Rev. 2021, 175, 113825. 10.1016/j.addr.2021.06.002 34111467

[2] R. Kaur , S. Arora , M. Goswami , Mater. Today: Proc. 2022, 2214, 10.1016/j.matpr.2022.11.182.

[3] M. R. Prausnitz , Adv. Drug Delivery Rev. 2004, 56, 581. 10.1016/j.addr.2003.10.023 15019747

[4] U. Angkawinitwong , A. J. Courtenay , A. M. Rodgers , E. Larrañeta , H. O. Mccarthy , S. Brocchini , R. F. Donnelly , G. R. Williams , ACS Appl. Mater. Interfaces 2020, 12, 12478. 32066234 10.1021/acsami.9b22425

[5] Q. K. Anjani , A. H. Bin Sabri , A. J. Hutton , Á. Cárcamo‐Martínez , L. A. H. Wardoyo , A. Z. Mansoor , R. F. Donnelly , J. Controlled Release 2023, 359, 97. 10.1016/j.jconrel.2023.05.038 37263545

[6] L. K. Vora , A. H. Sabri , Y. Naser , A. Himawan , A. R. J. Hutton , Q. K. Anjani , F. Volpe‐Zanutto , D. Mishra , M. Li , A. M. Rodgers , A. J. Paredes , E. Larrañeta , R. R. S. Thakur , R. F. Donnelly , Adv. Drug Delivery Rev. 2023, 201, 115055. 10.1016/j.addr.2023.115055 37597586

[7] J. M. Abu Ershaid , L. K. Vora , F. Volpe‐Zanutto , A. H. Sabri , K. Peng , Q. K. Anjani , P. E. McKenna , A. Ripolin , E. Larrañeta , H. O. McCarthy , R. F. Donnelly , Biomater. Adv. 2023, 153, 213526. 37348183 10.1016/j.bioadv.2023.213526

[8] F. Volpe‐Zanutto , L. K. Vora , I. A. Tekko , P. E. McKenna , A. D. Permana , A. H. Sabri , Q. K. Anjani , H. O. McCarthy , A. J. Paredes , R. F. Donnelly , J. Controlled Release 2022, 348, 771. 10.1016/j.jconrel.2022.06.028 35738464

[9] F. Chen , Q. Yan , Y. Yu , M. X. Wu , J. Controlled Release 2017, 255, 36. 10.1016/j.jconrel.2017.03.397 PMC6091871 28390901

[10] Y. Li , H. Zhang , R. Yang , Y. Laffitte , U. Schmill , W. Hu , M. Kaddoura , E. J. M. Blondeel , B. Cui , Microsyst. Nanoeng. 2019, 5, 41. 31636931 10.1038/s41378-019-0077-y PMC6799813

[11] L. K. Vora , A. H. Sabri , P. E. McKenna , A. Himawan , A. R. J. Hutton , U. Detamornrat , A. J. Paredes , E. Larrañeta , R. F. Donnelly , Nat. Rev. Bioeng. 2023, 2, 64.

[12] R. F. Donnelly , M. T. C. McCrudden , A. Z. Alkilani , E. Larrañeta , E. McAlister , A. J. Courtenay , M. C. Kearney , R. R. S. Thakur , H. O. McCarthy , V. L. Kett , E. Caffarel‐Salvador , S. Al‐Zahrani , A. D. Woolfson , PLoS One 2014, 9, 1. 10.1371/journal.pone.0111547 PMC4216095 25360806

[13] Q. K. Anjani , A. D. Permana , Á. Cárcamo‐Martínez , J. Domínguez‐Robles , I. A. Tekko , E. Larrañeta , L. K. Vora , D. Ramadon , R. F. Donnelly , Eur. J. Pharm. Biopharm. 2021, 294–312, 294. 10.1016/j.ejpb.2020.12.003 33309844

[14] S. Demartis , Q. K. Anjani , F. Volpe‐Zanutto , A. J. Paredes , S. A. Jahan , L. K. Vora , R. F. Donnelly , E. Gavini , Int. J. Pharm. 2022, 627, 122217. 36155790 10.1016/j.ijpharm.2022.122217

[15] Q. K. Anjani , A. K. Pandya , S. Demartis , J. Domínguez‐Robles , N. Moreno‐Castellanos , H. Li , E. Gavini , V. B. Patravale , R. F. Donnelly , Int. J. Pharm. 2023, 646, 123446. 37751787 10.1016/j.ijpharm.2023.123446

[16] E. Altuntaş , I. A. Tekko , L. K. Vora , N. Kumar , R. Brodsky , O. Chevallier , E. McAlister , Q. Kurnia Anjani , H. O. McCarthy , R. F. Donnelly , Int. J. Pharm. 2022, 614, 121422. 34958899 10.1016/j.ijpharm.2021.121422

[17] K. Peng , L. K. Vora , J. Domínguez‐Robles , Y. A. Naser , M. Li , E. Larrañeta , R. F. Donnelly , Mater. Sci. Eng., C 2021, 127, 112226. 10.1016/j.msec.2021.112226 34225871

[18] E. H. Mojumdar , L. B. Madsen , H. Hansson , I. Taavoniku , K. Kristensen , C. Persson , A. K. Morén , R. Mokso , A. Schmidtchen , T. Ruzgas , J. Engblom , Biomedicines 2021, 9, 360. 33807251 10.3390/biomedicines9040360 PMC8065685

[19] E. M. Vicente‐Perez , E. Larrañeta , M. T. C. McCrudden , A. Kissenpfennig , S. Hegarty , H. O. McCarthy , R. F. Donnelly , Eur. J. Pharm. Biopharm. 2017, 117, 400. 28478160 10.1016/j.ejpb.2017.04.029 PMC5496649

[20] R. Al‐Kasasbeh , A. J. Brady , A. J. Courtenay , E. Larrañeta , M. T. C. McCrudden , D. O'Kane , S. Liggett , R. F. Donnelly , Drug Delivery Transl. Res. 2020, 10, 690. 10.1007/s13346-020-00727-2 PMC7228965 32103450

[21] T. Y. Kuo , C. C. Huang , S. J. Shieh , Y. Bin Wang , M. J. Lin , M. C. Wu , L. L. H. Huang , Sci. Rep. 2022, 12, 445. 35013386 10.1038/s41598-021-03855-y PMC8748672

[22] D. T. Schomberg , A. Tellez , J. J. Meudt , D. A. Brady , K. N. Dillon , F. K. Arowolo , J. Wicks , S. D. Rousselle , D. Shanmuganayagam , Toxicol. Pathol. 2016, 44, 299. 26839324 10.1177/0192623315618292

[23] T. Käser , Mol. Immunol. 2021, 135, 95. 33873098 10.1016/j.molimm.2021.04.004

[24] W. Li , S. Li , X. Fan , M. R. Prausnitz , J. Controlled Release 2021, 339, 350. 10.1016/j.jconrel.2021.09.036 34597745

[25] J. Hackethal , F. Iredahl , J. Henricson , C. D. Anderson , E. Tesselaar , Skin Res. Technol. 2021, 27, 121. 32662126 10.1111/srt.12918

[26] M. B. McGuckin , A. R. J. Hutton , E. R. Davis , A. H. B. Sabri , A. Ripolin , A. Himawan , Y. A. Naser , R. Ghanma , B. Greer , H. O. McCarthy , A. J. Paredes , E. Larrañeta , R. F. Donnelly , Mol. Pharmaceutics 2024, 21, 2512. 10.1021/acs.molpharmaceut.4c00065 PMC11080471 38602861

[27] D. Reyna , I. Bejster , A. Chadderdon , C. Harteg , Q. Kurnia Anjani , A. Hidayat Bin Sabri , A. N. Brown , G. L. Drusano , J. Westover , E. Bart Tarbet , L. K. Vora , R. F. Donnelly , E. Lipka , Int. J. Pharm. 2023, 641, 123081. 37230371 10.1016/j.ijpharm.2023.123081 PMC10347771

[28] R. F. Donnelly , T. R. R. Singh , M. J. Garland , K. Migalska , R. Majithiya , C. M. McCrudden , P. L. Kole , T. M. T. Mahmood , H. O. McCarthy , A. D. Woolfson , Adv. Funct. Mater. 2012, 22, 4879. 23606824 10.1002/adfm.201200864 PMC3627464

[29] Q. K. Anjani , A. R. J. Hutton , A. H. Bin Sabri , F. Annuryanti , H. O. McCarthy , R. F. Donnelly , Biomater. Adv. 2025, 176, 214343. 40382893 10.1016/j.bioadv.2025.214343

[30] Q. K. Anjani , A. H. Bin Sabri , E. Utomo , J. Domínguez‐Robles , R. F. Donnelly , Mol. Pharmaceutics 2022, 19, 1191. 10.1021/acs.molpharmaceut.1c00988 PMC9097526 35235330

[31] Y. A. Naser , I. A. Tekko , L. K. Vora , K. Peng , Q. K. Anjani , B. Greer , C. Elliott , H. O. McCarthy , R. F. Donnelly , J. Controlled Release 2023, 356, 416. 10.1016/j.jconrel.2023.03.003 36878320

[32] L. Li , Q. K. Anjani , A. R. J. Hutton , M. Li , A. H. Bin Sabri , L. Vora , Y. A. Naser , Y. Tao , H. O. McCarthy , R. F. Donnelly , Drug Delivery Transl. Res. 2024, 1, https://link.springer.com/article/10.1007/s13346‐024‐01737‐0. 10.1007/s13346-024-01737-0 PMC12137397 39565514

[33] L. Zhao , L. K. Vora , S. A. Kelly , L. Li , E. Larrañeta , H. O. McCarthy , R. F. Donnelly , J. Controlled Release 2023, 356, 196. 10.1016/j.jconrel.2023.02.031 PMC7618114 36868520

[34] I. A. Tekko , L. K. Vora , F. Volpe‐Zanutto , K. Moffatt , C. Jarrahian , H. O. McCarthy , R. F. Donnelly , Adv. Funct. Mater. 2022, 32, 2106999.

[35] Q. K. Anjani , S. Demartis , N. Moreno‐Castellanos , E. Gavini , R. F. Donnelly , J. Pharm. Invest. 2024, 54, 683.

[36] Q. K. Anjani , A. H. Bin Sabri , K. A. Hamid , N. Moreno‐Castellanos , H. Li , R. F. Donnelly , Carbohydr. Polym. 2023, 320, 121194. 37659788 10.1016/j.carbpol.2023.121194

[37] Q. K. Anjani , A. Hidayat , B. Sabri , N. Moreno‐Castellanos , E. Utomo , Á. Cárcamo‐Martínez , J. Domínguez‐Robles , L. Ahmadi , H. Wardoyo , R. F. Donnelly , Biomater. Sci. 2022, 10, 5838. 35972236 10.1039/d2bm01068b

[38] Q. K. Anjani , F. Volpe‐Zanutto , K. A. Hamid , A. H. Bin Sabri , N. Moreno‐Castellano , X. A. Gaitán , J. Calit , D. Y. Bargieri , R. F. Donnelly , J. Controlled Release 2023, 361, 385. 10.1016/j.jconrel.2023.08.009 37562555

[39] A. S. Cordeiro , I. A. Tekko , M. H. Jomaa , L. Vora , E. McAlister , F. Volpe‐Zanutto , M. Nethery , P. T. Baine , N. Mitchell , D. W. McNeill , R. F. Donnelly , Pharm. Res. 2020, 37, 174. 32856172 10.1007/s11095-020-02887-9 PMC7452932

[40] A. H. Bin Sabri , Q. K. Anjani , E. Utomo , A. Ripolin , R. F. Donnelly , Int. J. Pharm. 2022, 617, 121593. 35182702 10.1016/j.ijpharm.2022.121593

[41] A. M. Abraham , Q. K. Anjani , M. Adhami , A. R. J. Hutton , E. Larrañeta , R. F. Donnelly , J. Mater. Chem. B 2024, 12, 4375. 38477350 10.1039/d4tb00110a

[42] Q. K. Anjani , N. Moreno‐Castellanos , Y. Li , A. H. Bin Sabri , R. F. Donnelly , Eur. J. Pharm. Biopharm. 2024, 199, 114304. 38663522 10.1016/j.ejpb.2024.114304

[43] Q. K. Anjani , N. Moreno‐Castellanos , M. Adhami , D. Ramadon , J. Jangga , R. F. Donnelly , Drug Delivery Transl. Res. 2024, 15, 355. 10.1007/s13346-024-01616-8 PMC11614984 38722459

[44] Q. K. Anjani , A. H. Bin Sabri , M. B. McGuckin , H. Li , K. A. Hamid , R. F. Donnelly , AAPS PharmSciTech 2022, 23, 273. 36195761 10.1208/s12249-022-02422-6

[45] Q. K. Anjani , Á. Cárcamo‐Martínez , L. A. H. Wardoyo , N. Moreno‐Castellanos , A. H. Bin Sabri , E. Larrañeta , R. F. Donnelly , Drug Delivery Transl. Res. 2023, 14, 208. 10.1007/s13346-023-01393-w PMC10746783 37477867

[46] Q. Yan , S. Shen , Y. Wang , J. Weng , A. Wan , G. Yang , L. Feng , Pharmaceutics 2022, 14, 1625. 36015251 10.3390/pharmaceutics14081625 PMC9413279

[47] W. Shu , H. Heimark , N. Bertollo , D. J. Tobin , E. D. O'Cearbhaill , A. N. Annaidh , Acta Biomater. 2021, 135, 403. 34492370 10.1016/j.actbio.2021.08.045

[48] Q. K. Anjani , A. D. C. Nainggolan , H. Li , A. Miatmoko , E. Larrañeta , R. F. Donnelly , Int. J. Pharm. 2024, 655, 124071. 38554738 10.1016/j.ijpharm.2024.124071

[49] W. Li , J. Tang , R. N. Terry , S. Li , A. Brunie , R. L. Callahan , R. K. Noel , C. A. Rodríguez , S. P. Schwendeman , M. R. Prausnitz , Sci. Adv. 2019, 5, 1. 10.1126/sciadv.aaw8145 PMC6834388 31723599

[50] E. H. Mojumdar , L. B. Madsen , H. Hansson , I. Taavoniku , K. Kristensen , C. Persson , A. K. Morén , R. Mokso , A. Schmidtchen , T. Ruzgas , J. Engblom , Biomedicines 2021, 9, 360. 33807251 10.3390/biomedicines9040360 PMC8065685

[51] N. K. Brogden , M. Milewski , P. Ghosh , L. Hardi , L. J. Crofford , A. L. Stinchcomb , J. Controlled Release 2012, 163, 220. 10.1016/j.jconrel.2012.08.015 PMC3725617 22929967

[52] G. Grubauer , P. M. Elias , K. R. Feingold , J. Lipid Res. 1989, 30, 323. 2723540

[53] S. L. Banks , K. S. Paudel , N. K. Brogden , C. D. Loftin , A. L. Stinchcomb , Pharm. Res. 2011, 28, 1211. 21301935 10.1007/s11095-011-0372-2 PMC3377386

[54] D. P. Wermeling , S. L. Banks , D. A. Hudson , H. S. Gill , J. Gupta , M. R. Prausnitz , A. L. Stinchcomb , Proc. Natl. Acad. Sci. U S A 2008, 105, 2058. 18250310 10.1073/pnas.0710355105 PMC2538880

[55] J. H. Park , J. W. Lee , Y. C. Kim , M. R. Prausnitz , Int. J. Pharm. 2008, 359, 94. 18455889 10.1016/j.ijpharm.2008.03.032 PMC2464624

[56] G. Honari , H. Maibach , Appl. Dermatotoxicol.: Clin. Aspects 2014, 1.

[57] H. Kalluri , A. K. Banga , Pharm. Res. 2011, 28, 82, https://link.springer.com/rwe/10.1007/978‐3‐319-68617‐2_104. 20354766 10.1007/s11095-010-0122-x

[58] A. G. Harris , C. Naidoo , D. F. Murrell , Int. J. Womens Dermatol. 2015, 1, 77. 28491962 10.1016/j.ijwd.2015.03.004 PMC5418754

[59] A. Futagami , M. Ishizaki , Y. Fukuda , S. Kawana , N. Yamanaka , Lab. Invest. 2002, 82, 1503. 12429810 10.1097/01.lab.0000035024.75914.39

[60] S. L. Banks , R. R. Pinninti , H. S. Gill , K. S. Paudel , P. A. Crooks , N. K. Brogden , M. R. Prausnitz , A. L. Stinchcomb , J. Pharm. Sci. 2010, 99, 3072. 20166200 10.1002/jps.22083 PMC2862091

[61] H. Kalluri , A. K. Banga , Pharm. Res. 2011, 28, 82. 20354766 10.1007/s11095-010-0122-x

[62] P. M. Elias , J. S. Wakefield , Dermatologica Sin. 2015, 33, 49.

[63] R. F. Donnelly , T. R. R. Singh , M. M. Tunney , D. I. J. Morrow , P. A. McCarron , C. O'Mahony , A. D. Woolfson , Pharm. Res. 2009, 26, 2513. 19756972 10.1007/s11095-009-9967-2 PMC2900181

[64] T. M. Hooton , D. Mandell , Bennett Princ. Pract. Infect. Dis. 2015, 2, 3334.

[65] J. O. N. Lundberg , S. Carlsson , L. Engstrand , E. Morcos , N. P. Wiklund , E. Weitzberg , Urology 1997, 50, 189. 9255286 10.1016/S0090-4295(97)00257-4

[66] Urine Glucose Test: Purpose, Procedure, and Results , https://www.healthline.com/health/glucose‐test‐urine (accessed: April 2025).

[67] Urobilinogen in Urine – What is Urobilinogen in Urine , https://massivebio.com/urobilinogen‐in‐urine/ (accessed: January 2025).

[68] Assessment of proteinuria – Differential diagnosis of symptoms | BMJ Best Practice , https://bestpractice.bmj.com/topics/en‐gb/875 (accessed: January 2025).

[69] “Specific Gravity of Urine” can be found under https://my.clevelandclinic.org/health/diagnostics/specific-gravity-of-urine (accessed: April 2025).

[70] Creatinine Urine Test: Understanding the Test and Results , https://www.healthline.com/health/creatinine‐clearance#results (accessed: January 2025).

[71] Ketones urine test , https://www.ucsfhealth.org/medical‐tests/ketones‐urine‐test (accessed: January 2025).

[72] İ. Yıldırım , H. Koçan , Cureus 2023, 15, 47437. 10.7759/cureus.47437 PMC10659234 38022142

[73] P. Chen , B. Sun , H. Chen , G. Wang , S. Pan , R. Kong , X. Bai , S. Wang , Cytokine 2010, 49, 15. 19900821 10.1016/j.cyto.2009.09.013

[74] T. A. E. Platts‐Mills , Am J. Respir. Crit. Care Med. 2001, 164, 1107. 11673190 10.1164/ajrccm.164.7.2107130b

[75] S. T. Al‐Otaibi , H. A. M. Alqahtani , J. Dermatol. Dermatol. Surg. 2015, 19, 86.

[76] G. Vidarsson , G. Dekkers , T. Rispens , Front. Immunol. 2014, 5, 520. 25368619 10.3389/fimmu.2014.00520 PMC4202688

[77] J. Charles A Janeway , P. Travers , M. Walport , M. J. Shlomchik , 2001.

[78] L. Simon , F. Gauvin , D. K. Amre , P. Saint‐Louis , J. Lacroix , Clin. Infect. Dis. 2004, 39, 206. 15307030 10.1086/421997

[79] M. A. Mendall , P. Patel , L. Ballam , D. Strachan , T. C. Northfield , BMJ 1996, 312, 1061. 8616412 10.1136/bmj.312.7038.1061 PMC2350910

[80] S. Black , I. Kushner , D. Samols , J. Biol. Chem. 2004, 279, 48487, https://www.ncbi.nlm.nih.gov/books/NBK27162/. 15337754 10.1074/jbc.R400025200

[81] T. W. Du Clos , Ann. Med. 2000, 32, 274. 10852144 10.3109/07853890009011772

[82] N. R. Sproston , J. J. Ashworth , Front. Immunol. 2018, 9, 754. 29706967 10.3389/fimmu.2018.00754 PMC5908901

[83] G. Lopez‐Castejon , D. Brough , Cytokine Growth Factor Rev. 2011, 22, 189. 22019906 10.1016/j.cytogfr.2011.10.001 PMC3714593

[84] Y. Cai , F. Xue , C. Quan , M. Qu , N. Liu , Y. Zhang , C. Fleming , X. Hu , H. ge Zhang , R. Weichselbaum , Y. X. Fu , D. Tieri , E. C. Rouchka , J. Zheng , J. Yan , J. Invest. Dermatol. 2019, 139, 146. 30120937 10.1016/j.jid.2018.07.025 PMC6392027

[85] Q. K. Anjani , U. Detamornrat , E. Larrañeta , R. F. Donnelly , Int. J. Pharm. 2025, 669, 125061. 39653288 10.1016/j.ijpharm.2024.125061

[86] A. H. Bin Sabri , Q. K. Anjani , P. Gurnani , J. Domínguez‐Robles , N. Moreno‐Castellanos , L. Zhao , A. R. J. Hutton , R. F. Donnelly , Int. J. Pharm. 2023, 644, 123292. 37553057 10.1016/j.ijpharm.2023.123292

[87] Q. K. Anjani , A. H. Bin Sabri , J. Domínguez‐Robles , N. Moreno‐Castellanos , E. Utomo , L. A. H. Wardoyo , E. Larrañeta , R. F. Donnelly , Biomater. Adv. 2022, 140, 213073. 35964387 10.1016/j.bioadv.2022.213073

[88] E. Larraneta , J. Moore , E. M. Vicente‐Perez , P. Gonzalez‐Vazquez , R. Lutton , A. D. Woolfson , R. F. Donnelly , Int. J. Pharm. 2014, 472, 65. 24877757 10.1016/j.ijpharm.2014.05.042 PMC4111867

[89] D. T. Schomberg , A. Tellez , J. J. Meudt , D. A. Brady , K. N. Dillon , F. K. Arowolo , J. Wicks , S. D. Rousselle , D. Shanmuganayagam , Toxicol. Pathol. 2016, 44, 299. 26839324 10.1177/0192623315618292

[90] E. Kobayashi , S. Hishikawa , T. Teratani , A. T. Lefor , Transplant. Res. 2012, 1, 8. 23369409 10.1186/2047-1440-1-8 PMC3560993

[91] A. Stricker‐Krongrad , C. R. Shoemake , M. E. Pereira , S. C. Gad , D. Brocksmith , G. F. Bouchard , Sage J. 2015, 44, 421. 10.1177/0192623315613337 26656239

[92] D. K. Meyerholz , E. R. Burrough , N. Kirchhof , D. J. Anderson , K. L. Helke , Sage J. 2024, 61, 512. 10.1177/03009858231222235 38197394

