# Supplementary material for: Safety Evaluation of Repeated Application of Polymeric Microarray Patches in Miniature Pigs
Source: Adv Healthc Mater. 2025 Jun 17;14(22):2501512. doi: 10.1002/adhm.202501512 (PMC12391629; doi:10.1002/adhm.202501512)
Supplement: Supplementary file 1 — Supporting Information [file ADHM-14-0-s001.docx]

**Supplementary data**

**Safety Evaluation of Repeated Application of Polymeric Microarray Patches in Miniature Pigs**

*Qonita Kurnia Anjani^1^, Aaron R. J. Hutton^2^, Peter E. McKenna^1^, Eneko Larrañeta^1^, Ryan F. Donnelly^1,*^*

*Corresponding author: Ryan F. Donnelly ([r.donnelly@qub.ac.uk](mailto:r.donnelly@qub.ac.uk))

1. School of Pharmacy, Queen’s University Belfast, Medical Biology Centre, 97 Lisburn Road, Belfast BT9 7BL, UK.
2. School of Pharmacy and Pharmaceutical Sciences, Ulster University, Pharmacy Building, Block Y, 1SA, Cromore Rd, Coleraine BT52 1SA, UK

Table S1. Summary of the statistical significance obtained by comparing the different MAP evaluated over the days. Several significant differences between the different study areas were found (Covered-MAP, Covered-no MAP, Uncovered-no MAP). The significance level is shown as: * P ≤ 0.05, ** P ≤ 0.01 and *** P ≤0.001.

|  |  |  |  | | **Dissolving MAP** | | | **Implantable MAP** | | | **Hydrogel-forming MAP** | |
| --- | --- | --- | --- | --- | --- | --- | --- | --- | --- | --- | --- | --- |
| **Study day** | **Comparisons** | | | P-Value | | Significance Summary | P-Value | | Significance Summary | P-Value | | Significance Summary |
| D0 | Covered-MAP vs. Covered-no MAP | | | 0.5565 | | ns | 0.0165 | | * | 0.8552 | | ns |
|  | Covered-MAP vs. Uncovered-no MAP | | | 0.3557 | | ns | 0.0102 | | * | 0.8251 | | ns |
|  | Covered-no MAP vs. Uncovered-no MAP | | | 0.9378 | | ns | 0.9858 | | ns | 0.9982 | | ns |
| D2 | Covered-MAP vs. Covered-no MAP | | | 0.0008 | | *** | 0.9996 | | ns |  | | |
|  | Covered-MAP vs. Uncovered-no MAP | | | 0.1906 | | ns | 0.9889 | | ns |  |  |  |
|  | Covered-no MAP vs. Uncovered-no MAP | | | 0.1337 | | ns | 0.9841 | | ns |  |  |  |
| D4 | Covered-MAP vs. Covered-no MAP | | |  | | |  | | | 0.0462 | | * |
|  | Covered-MAP vs. Uncovered-no MAP | | |  |  |  |  |  |  | 0.4859 | | ns |
|  | Covered-no MAP vs. Uncovered-no MAP | | |  |  |  |  |  |  | 0.4307 | | ns |
| D7 | Covered-MAP vs. Covered-no MAP | | | 0.5506 | | ns | 0.007 | | ** | 0.0746 | | ns |
|  | Covered-MAP vs. Uncovered-no MAP | | | 0.0326 | | * | <0.0001 | | **** | 0.0002 | | *** |
|  | Covered-no MAP vs. Uncovered-no MAP | | | 0.3026 | | ns | 0.3506 | | ns | 0.1577 | | ns |
| D9 | Covered-MAP vs. Covered-no MAP | | | 0.4803 | | ns | 0.1007 | | ns |  | | |
|  | Covered-MAP vs. Uncovered-no MAP | | | >0.9999 | | ns | 0.7403 | | ns |  |  |  |
|  | Covered-no MAP vs. Uncovered-no MAP | | | 0.4803 | | ns | 0.3865 | | ns |  |  |  |
| D11 | Covered-MAP vs. Covered-no MAP | | |  | | |  | | | 0.0002 | | *** |
|  | Covered-MAP vs. Uncovered-no MAP | | |  |  |  |  |  |  | 0.9988 | | ns |
|  | Covered-no MAP vs. Uncovered-no MAP | | |  |  |  |  |  |  | 0.0002 | | *** |
| D14 | Covered-MAP vs. Covered-no MAP | | | <0.0001 | | **** | 0.0028 | | ** | 0.2527 | | ns |
|  | Covered-MAP vs. Uncovered-no MAP | | | <0.0001 | | **** | 0.6464 | | ns | 0.0235 | | * |
|  | Covered-no MAP vs. Uncovered-no MAP | | | 0.9929 | | ns | 0.0408 | | * | 0.5432 | | ns |
| D16 | Covered-MAP vs. Covered-no MAP | | | 0.1008 | | ns | 0.5506 | | ns |  | | |
|  | Covered-MAP vs. Uncovered-no MAP | | | 0.8062 | | ns | 0.2306 | | ns |  |  |  |
|  | Covered-no MAP vs. Uncovered-no MAP | | | 0.021 | | * | 0.021 | | * |  |  |  |
| D18 | Covered-MAP vs. Covered-no MAP | | |  | | |  | | | 0.0094 | | ** |
|  | Covered-MAP vs. Uncovered-no MAP | | |  |  |  |  |  |  | 0.3533 | | ns |
|  | Covered-no MAP vs. Uncovered-no MAP | | |  |  |  |  |  |  | <0.0001 | | **** |
| D21 | Covered-MAP vs. Covered-no MAP | | | 0.0062 | | ** | 0.0471 | | * | 0.493 | | ns |
|  | Covered-MAP vs. Uncovered-no MAP | | | 0.0556 | | ns | 0.0763 | | ns | 0.918 | | ns |
|  | Covered-no MAP vs. Uncovered-no MAP | | | 0.7173 | | ns | 0.9784 | | ns | 0.2786 | | ns |
| D23 | Covered-MAP vs. Covered-no MAP | | | 0.4132 | | ns | 0.0012 | | ** |  | | |
|  | Covered-MAP vs. Uncovered-no MAP | | | 0.9202 | | ns | 0.0152 | | * |  |  |  |
|  | Covered-no MAP vs. Uncovered-no MAP | | | 0.6524 | | ns | 0.7231 | | ns |  |  |  |
| D25 | Covered-MAP vs. Covered-no MAP | | |  | | |  | | | 0.9988 | | ns |
|  | Covered-MAP vs. Uncovered-no MAP | | |  |  |  |  |  |  | 0.739 | | ns |
|  | Covered-no MAP vs. Uncovered-no MAP | | |  |  |  |  |  |  | 0.7665 | | ns |
| D28 | Covered-MAP vs. Covered-no MAP | | | 0.9125 | | ns | <0.0001 | | **** | 0.2629 | | ns |
|  | Covered-MAP vs. Uncovered-no MAP | | | 0.5864 | | ns | <0.0001 | | **** | 0.0125 | | * |
|  | Covered-no MAP vs. Uncovered-no MAP | | | 0.3457 | | ns | 0.4688 | | ns | 0.3976 | | ns |

*Significance level: * P ≤ 0.05, ** P ≤ 0.01 and *** P ≤0.001, ns: no significant*
